# Nutrient-Driven Modulation of Microbial, Plant, and Rhizosphere Processes for Heavy Metal Remediation

**DOI:** 10.3390/plants15101517

**Published:** 2026-05-15

**Authors:** Lixia Wang, Xiaoping Zang, Hafiz Faiq Bakhat, Ghulam Abbas Shah, Tao Jing, Yan Zhao, Yingdui He

**Affiliations:** 1 Key Laboratory of Banana Genetic Improvement of Hainan Province, Institute of Tropical Bioscience and Biotechnology, Sanya Research Institute, Chinese Academy of Tropical Agricultural Sciences, Haikou 571101, China; wanglixia@catasitbb.cn (L.W.); zangxiaoping@catasitbb.cn (X.Z.); faiqsiddique@ciitvehari.edu.pk (H.F.B.); shahga@uaar.edu.pk (G.A.S.); jingtao@catasitbb.cn (T.J.); 2 Department of Environmental Sciences, COMSATS University Islamabad, Vehari Campus, Islamabad 61100, Pakistan; 3 Department of Agronomy, Pir Mehr Ali Shah Arid Agriculture University Rawalpindi, Rawalpindi 46300, Pakistan; 4 College of Tropical Agriculture and Forestry, Hainan University, Haikou 570228, China

**Keywords:** microbial processes, phytoextraction, rhizosphere, arbuscular mycorrhizal fungi, metal chelating agents

## Abstract

Heavy metal pollution remains a major global environmental challenge due to persistent ecological risks and potential threats to food safety. Microbial remediation and phytoremediation represent sustainable alternatives to conventional treatments; however, their effectiveness is strongly influenced by number of factors including nutrient availability. This review critically examines how nutritional regulation governs microbial metabolism, plant physiological responses, and rhizosphere interactions to enhance heavy metal transformation and removal. Metal bioavailability depends on type, concentration, soil pH, redox potential, and microbial processes. Interventions including fertilizers, chelating agents, inoculation with arbuscular mycorrhizal fungi and plant-growth-promoting rhizobacteria enhance phytoremediation processes through regulating plant nutrient and heavy metal uptake, while selection between ammonium/nitrate changes rhizosphere pH consequently affects plant metal uptake. Similarly, nutrients, i.e., phosphate, iron, zinc and manganese competitively affect metal uptake. Organic amendments enhance phytostabilization, especially for selenium and mercury, while enhancing chromium reduction. Sulfur-reducing bacteria precipitate metals as insoluble sulfides with 90% efficiency. In addition, soil amendments including plant-growth-promoting rhizobacteria, arbuscular mycorrhizal fungi, and metal-chelating agents can be strategically used to enhance the phytoextraction from metal from contaminated soils. We suggest that the future integration of modern approaches such as multi-omics and cisgenesis supported by artificial intelligence tools can help to accurately predict the efficiency of nutrient regulation strategies and their remediation outcomes, thereby supporting evidence-based soil management.

## 1. Introduction

**Figure 1:**
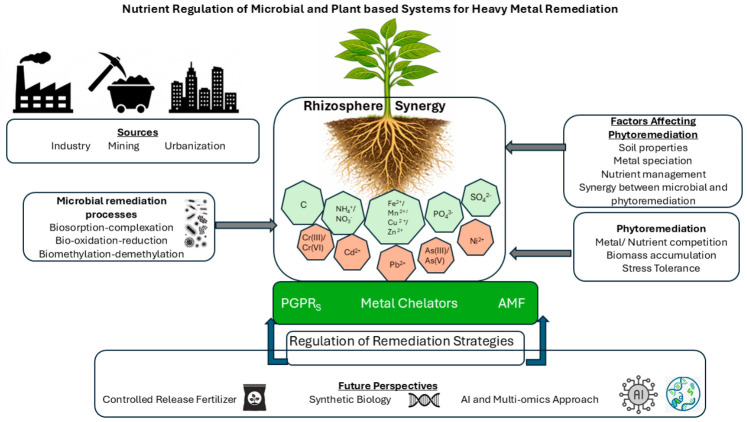
Conceptual framework showing the nutrient regulation of microbial- and plant-based systems for heavy metal remediation. Nutrient management regulates rhizosphere processes, including biosorption, redox transformation, and biomethylation. Rhizoremediation develops synergy between microbe and plant and improves phytoremediation efficiency. Emerging trend that can contribute to enhancing the efficiency of remediation processes includes controlled release fertilizers, synthetic biology approaches, and AI- and multi-omics-assisted optimization.

Global urbanization has reached a tipping point with a current 58% of the total population living in the cities as compared to 46.7% in 2000 [1]. This demographic shift, together with the rapid expansion of the global middle class, (to about 4 billion people), has accelerated industrialization and increased the Global Material Footprint from ~54 billion tons in 2000 to >100 billion tons in 2024 [2,3]. The industrialization and consumerism have led to widespread excavation and subsequent dumping of heavy metals to the ecosystem resulting in substantial pressure on global ecological functioning (Figure 1).

Pollutants released through natural and anthropogenic processes are transported and exchanged across various ecological spheres, such as lithosphere, hydrosphere, atmosphere, and biosphere. However, soil remains a unique medium in which all these spheres interact, and importantly, the medium provides approximately 95% of the food consumed by humans [4]. In addition, the increasing global population and improved living standards required to increase the food production by 35% to 56% by 2050 [5]. Soil quality and productivity are critically affected by heavy metals including cadmium (Cd), lead (Pb), chromium (Cr), nickel (Ni) and toxic metalloids arsenic (As), and mercury (Hg) owing to their non-degradable nature and long-term persistence in soil ecosystem [6]. Estimates suggest that around 20 million hectares of land is contaminated annually with heavy metals [7], potentially affecting 14% to 17% of global farmland [8].

Remediation of contaminated soil and protecting uncontaminated soil resources is crucial for sustaining global food security [9]. Chemical and physical remediation methods often disturb soil properties, produce toxic sludge, and involve high operational costs [10]. In contrast, bioremediation provides an environmentally friendly and sustainable alternative by utilizing the inherent abilities of microorganisms and plants to remove or transform pollutants [11]. However, its efficacy for widespread adoption is hindered by several challenges, including slow removal rates, site heterogeneity, chemical analogy of the heavy metals with plant essential nutrients and complexity of contaminants. Addressing these fundamental limitations requires optimizing the biological processes at their core. To enhance the efficiency of the bioremediation processes, specific strategies are adopted including stimulation of native microbial activities by adding nutrients and/or oxygen as well as introducing more efficient and specialized microbial species or consortia. Similarly, the efficacy of plants can be enhanced by optimizing the condition for growth and mineral nutrient availability and acquisition. In both microbe-mediated and plant-based remediation, the availability of mineral nutrients is critically important for improving remediation efficacy [12]. However, under natural conditions, the toxic heavy metals/metalloids (e.g., Cd, Pb and As), with chemical analogy to essential nutrients, can have competitive effect on their uptake and impair metabolic functioning of both microbes and plants [13].

This review emphasizes the importance of mineral nutrients and their regulation to enhance bioremediation effectiveness under heavy metal/metalloid stress. Furthermore, it explains the complex interactions between essential nutrients acquisition and heavy metal contamination by detailing the negative effects on the functioning of the biosystems. By elaborating these mechanisms, this article provides a mechanistic framework for integrating multiple remediation methods and employs genetic engineering with multi-omic approaches in real-world heavy metal cleanup efforts. Furthermore, advanced AI tools provide opportunities to identify “Gene Networks” responsible for metal uptake, translocation, detoxification, and transformation, revealing non-obvious targets for breeding to tackle the issue at larger scale.

## 2. Factors Influencing the Effectiveness of Bioremediation

### 2.1. Heavy Metal Properties

The type of heavy metal dictates the primary mechanism of cellular responses in microbes and plants; however, metal concentration and its chemical species play a vital role in its bioavailability and toxicity [14]. The most toxic metals, commonly referred to as priority pollutants, including Cd, Pb, Hg, As, Ni, Cr, and to a lesser extent copper (Cu), zinc (Zn), cobalt (Co), and manganese (Mn), are the concerns for ecosystem functioning. In addition to metal type, the bioavailable fraction of metals readily crosses cellular membranes and is considered the most toxic form, adversely affecting biological systems and the biosphere [15]. For instance, Cr(VI) is highly soluble, mutagenic, and toxic, while the Cr(III) is much less mobile and significantly less toxic in the biological systems [16]. Similarly, the organic species of As are less toxic than the inorganic species, i.e., arsenite As(III) and arsenate As(V) [17]. The effectiveness of nutritional regulation is therefore species-dependent (e.g., phosphate ion competes with As(V) and inhibits its uptake) [18]. Henceforth, assessing bioavailable fraction from the total metal concentration is crucial to avoid overestimation of risks associated with the metals.

The concentration of heavy metals determines whether the biological response is sub-lethal and chronic or acute and lethal. Long-term exposure to low levels of heavy metals leads to bioaccumulation in organism tissues (e.g., liver, kidney) and biomagnification across the food chain, resulting in chronic pathological effects [19]. Sub-lethal concentrations trigger molecular and biochemical responses (e.g., oxidative stress, enzyme inhibition) before observable ecological effects (mortality or reproductive failure). Therefore, heavy metal mitigation strategies are primarily aimed at supporting the early-stage cellular defenses [20].

In soil and other environmental matrices, heavy metals co-exist (e.g., Cd^2+^ and Pb^2+^), or a toxic metal may occur alongside elevated concentrations of an essential metal like Zn^2+^, which share the same membrane transporter. Under such conditions, toxicity often becomes synergistic, as simultaneous exposure rapidly oversaturates the plant glutathione (GSH) reserves, leaving the organisms defenseless against both metals and other oxidative stressors. Many heavy metals (e.g., Fe^2+^, Cu^2+^, Cd^2+^) induce oxidative stress by generating Reactive Oxygen Species (ROS). Simultaneous exposure to multiple redox-active metals produces additive ROS burden, rapidly depleting the cell antioxidant defenses, including Catalase (CAT) and Superoxide dismutase (SOD), thereby increasing the toxicity.

### 2.2. Soil Properties

The efficacy of nutritional regulation strategies to mitigate heavy metal toxicity is critically dependent upon soil properties. Soil pH fundamentally controls the speciation, adsorption and solubility of heavy metals [15]. Under acidic conditions, high H^+^ concentrations compete strongly with metal cations for binding sites on clays and organic matter, increasing metal ions’ soluble/bioavailable fraction. For instance, Cd^2+^ concentration significantly increases below pH 6.5 [21]. Conversely, high pH favors the sorption of metals at negative surfaces and promotes the precipitation of metals at metal-hydroxides or carbonates such as Pb typically precipitates as Pb(OH)_2_ or PbCO_3_ at pH > 6.0 [22].

The redox (Eh) status of the soil controls the solubility and toxicity of heavy metal/metalloids, especially As, Hg and Cr. Under oxidizing conditions, Cr and As exists as (Cr(VI) and As(V) with higher mobility and toxicity [23]. Conversely, reduction leads to the formation of highly stable and insoluble metal sulfide precipitates (e.g., CdS or PbS), thereby sequestering the metals and significantly decreasing their bioavailability [24].

### 2.3. Biological Factors

Microbiome in the rhizosphere are considered the second genome of plants and play a crucial role in soil detoxification of heavy metals; however, their transformation efficiency depends on their inherent ability [25]. Certain microbes possess genes that facilitate heavy metal transformations, including reduction of toxic Cr(VI) to less toxic Cr(III) as well as methylation mechanisms associated with As detoxification [26,27]. The adaptability of microbial strains to specific environmental conditions, such as pH, Eh, and multi-heavy metal toxicity, is the key factor determining the success of bioremediation. Microbial consortia often prove more effective than single strains, as they can collectively tolerate complex stressors, thereby enhancing the heavy metal remediation supported by the nutritional regulation strategies [28].

Plant survival under elevated heavy metal concentrations relies on their specific strategies such as metal exclusion (avoidance) or metal accumulation/hyperaccumulation [29]. Plant species, with the characteristics to maintain low heavy metal concentrations in their above-ground tissues (shoots) by minimizing uptake and/or restricting its translocation primarily works at the root level and in the rhizosphere. Similar exclusion strategies, such as Al^3+^-activated malate/citrate exudation via ALMT and MATE transporters, have been comprehensively reviewed in the context of aluminum resistance [25]. Heavy metal exclusion at the root surface is achieved through two main mechanisms: (i) downregulation of non-selective or specific metal transporters, such as NRAMP, IRT, and ZIP, effectively minimizing the influx of toxic metals like Cd^2+^ [30], and (ii) active efflux pumps, often belonging to the P-type ATPase family, which actively exclude the metals like Cu^2+^ and Zn^2+^ into the apoplast or rhizosphere. Heavy metals that enter the root apoplast are often bound with the cell wall due to the negative charges on pectin and hemicellulose. Metals like Pb^2+^ and Cu^2+^ show high affinity for these sites, with the restricted movement towards the vascular tissues [31]. Furthermore, the exudation of H^+^ ions can alter the localized pH near the root surface, influencing the solubility and precipitation of the metals. For instance, increasing the rhizosphere pH generally promotes the precipitation of cationic metals as insoluble hydroxides, effectively excluding them from the plant [32]. The effectiveness of applying lime and phosphate fertilizer to mitigate heavy metal toxicity is strongly influenced by buffering capacity of the rhizosphere.

Root exudates, such as low-molecular-weight organic acids such as citrate, malate, and oxalate which act as chelators, form soluble complexes with metal ions (e.g., Cd^2+^, Pb^2+^) and increase their bioavailability [33] which may lead to metal hyperaccumulation in above-ground tissues. Plants detoxify these metals through metal chelators (e.g., phytochelatins (PCs), cysteine-rich peptides synthesized enzymatically by phytochelatin synthase bind to a wide range of metals forming high-affinity PC–metal complexes [34]. In addition, plants have metallothioneins (MTs) that are small, gene-encoded proteins with a high sulfur content capable of binding toxic metals like Cd^2+^ [35]. Nutritional regulation involving sulfur supplementation can enhance the precursor availability for both PCs and MTs, thereby reducing heavy metal toxicity. The primary mechanism for long-term detoxification and tolerance in hyperaccumulators is the active transport and sequestration of these PC–metal complexes into the vacuole. Heavy metal accumulation often leads an oxidative stress with the generation of reactive oxygen species (ROS). Tolerant plants efficiently detoxify these ROS through: (i) enzymatic antioxidants such as SOD, CAT, and Ascorbate Peroxidase (APX), and (ii) non-enzymatic antioxidants such as Ascorbate, Glutathione (GSH), and tocopherols.

### 2.4. Nutrient Management Strategies

Remediation of heavy-metal-contaminated soils requires well-targeted nutrient management strategies rather than routine agronomic practice, to achieve optimal bioremediation. For instance, nutritional regulation (e.g., specific carbon sources) is only effective when it stimulates the growth and activity of metabolically competitive and effective microbial strain [36], Similarly, choice between ammonium (NH_4_^+^) and nitrate (NO_3_^−^) fertilizers is a key factor affecting metal solubility and mobility in the soil. Ammonium nutrition results in localized acidification, with increased bioavailability of cationic metals [37]. In contrast, application of NO_3_^−^ leads to localized alkalinization with the enhanced phytostabilization of heavy metals as insoluble hydroxides and carbonates, thereby reducing their bioavailability [38]. The intricate interplay between nitrogen form and iron acquisition [39] directly impacts the expression of divalent metal transporters which can inadvertently facilitate Cd^2+^ influx under iron deficiency. Application of phosphate fertilizer to metal-contaminated soils forms stable, low-solubility mineral precipitates such as pyromorphite (Pb_5_(PO_4_)_3_Cl), thereby fixing the metal into a crystalline structure and reducing its bioavailability [40]. Applying a high dose of nutrients that have the same ionic radii and charges as toxic heavy metals can reduce metal uptake competitively at the root surface [41]. For instance, high calcium dosage competitively blocks the membrane transport channels that facilitate Cd^2+^ and Pb^2+^ uptake, thereby reducing metals influx [42]. Similarly, application of Zn fertilizers can mitigate Cd^2+^ toxicity in both plants and microbes [43]. On the other hand, elements that do not have a competitive effect on the uptake of essential plant nutrients can still be strategically used to enhance the plant’s intrinsic detoxification capacity. Among these elements, S increases metal chelation, enabling the biological systems to sequester heavy metals into the vacuole [44]. Similarly, Se, a cofactor for the antioxidant enzyme GSH-Peroxidase, is essential to reduce the oxidative stress induced by heavy metals [45]. Appropriate fertilization ensures the cellular antioxidant defenses, preventing ROS damage caused by redox-active metals [46].

The choice of fertilizers as well as their timing and spatial application must align with remediation objectives. For phytoextraction, soluble fertilizers must be applied during the peak plant growth stages to maximize heavy metal removal. In contrast, metal-immobilizing agents such as lime, phosphate, or biosolids should be applied homogeneously early in remediation process to maximize contact with metals and promote metal stabilization. To overcome the specific nutrient deficiency (e.g., Fe, S), foliar application can be more effective as it delivers the nutrients directly to the metabolic site [36].

## 3. Key Microbial Processes for Heavy Metal Remediation

### 3.1. Bio-Adsorption (Biosorption) and Bioaccumulation

Biosorption is a physicochemical process in which potentially toxic elements rapidly bind to charged ligands on the microbial cell wall [47]. The efficiency of metal biosorption depends mainly on the composition of the microbial cell wall and the functional groups in exopolysaccharides. For instance, fungal cell wall is rich in carboxyl (–COOH) and amino (–NH_2_) groups primarily due to the presence of chitin and glucans [48]. Metal biosorption on these polymers is highly pH-dependent, with competition occurring between metal cations and H^+^ ions for binding sites at lower pH values [49]. In contrast, algae cell wall is composed of polymers like alginates, fucoidan and carrageenans. Alginates have carboxylate (–COO^−^), a weak acid group deprotonated only above pH 4, while fucoidan and carrageenans have sulfonate and sulphate (–SO_3_^−^ or –OSO_3_^−^) which provide binding surfaces for metal cations across a wide pH range [11]. Peptidoglycan polymer in bacterial cell wall, particularly in Gram-positive bacteria, contains phosphate-rich teichoic acids (wall teichoic acids and lipoteichoic acids), in addition to carboxyl groups, making them highly suitable for biosorption of metals such as Pb^2+^ and Cd^2+^ [50]. Although biosorption is a passive process, exopolysaccharide production is both metabolically and genetically regulated [33]. Therefore, gene regulation by external factors such as nutrient supply can indirectly influence the biosorption potential of microbes [51].

Bioaccumulation is an active, metabolic-dependent process that requires the transport of metals across the plasma membrane into the cytoplasm. Metal ions enter the cell through various types of transport proteins including channels, carriers and pumps. Energy-dependent proteins on the cell surface, particularly P-type ATPases, use ATP to generate proton (H^+^) gradients that facilitate heavy metals uptake into the microbial cell [52]. For instance, Cr(VI) can enter cells via sulfate transporters, while As(V) can enter via phosphate transporters and aquaglyceroporins [53,54]. Similarly, Pb^2+^ and Cd^2+^ enter cells through divalent metal transporters like Mn^2+^ and Zn^2+^, or via Fe^2+^ transport-specific ligands like siderophores [55]. Certain microbes may regulate the efflux of a particular toxic metal to maintain the internal concentrations below toxic levels. Bacterial species like *Escherichia coli* and *Bacillus* possess As resistance operon (Ars), which regulates intercellular As level and activates ATP-dependent efflux pump (ArsB) [56]. In contrast, fungi and algae exposure to certain metals like Cd^2+^, Cu^2+^, Zn^2+^, Sb^3+^, Ag^+^, Ni^2+^, Hg^2+^ stimulates phytochelatin synthase enzyme activity at the transcription and translation level, promoting intracellular sequestration of these metals [57,58].

### 3.2. Bio-Oxidation and Reduction

Biotransformation processes cause a fundamental change in the valence state of the metal and modify its solubility, mobility and toxicity. Bio-reduction can transform toxic, mobile, oxidized species such as Cr(VI), Sb(V), and U(VI) into less toxic, insoluble, reduced species such as Cr(III), Sb(III) and U(IV) [27,59]. Under anaerobic conditions, microbes such as *Exiguobacterium* sp. PY14 and species of the genus *Lysinibacillus* use Cr(VI) and Sb(V) as terminal electron acceptors [60,61]. In *Pseudomonas* sp., the gene expression for chromate reductase (ChrR) is transcriptionally upregulated in the presence of Cr(VI), highlighting a direct genetic adaptation to the toxic metal [62].

Oxidation process increases the valence state such as conversion from As(III) to As(V) or Mn^2+^ to Mn^4+^, thereby affecting solubility, mobility and toxicity of heavy metals [60,63]. Arsenite oxidation is carried out by bacterial species through As(III)-oxidase enzyme, often coupled with electron transport chain to generate ATP. The As(III) oxidation is controlled by the aio operon whose expression is regulated by As levels and the availability of terminal electron acceptors, thereby linking As(III) detoxification directly to respiratory metabolism [19].

### 3.3. Biomethylation and Demethylation

Biomethylation and demethylation alter heavy metals and metalloid species by adding or removing methyl (CH_3_) groups, respectively [64]. Mercury methylation refers typically to the enzymatic transfer of a methyl group to Hg^2+^ via the hgcAB pathway [65]. The genes required for Hg methylation, i.e., *hgcA* and *hgcB*, are expressed exclusively under anoxic conditions. The methylation is not only controlled at physiological and biochemical level but also at the level of gene expression, e.g., high level of *hgcAB* expression in *Pseudodesulfovibrio mercurii* ND132 is associated with the Hg-methylation [66]. In contrast, some microbial species, including *Chloroflexi*, *Nitrospina*, *Verrucomicrobia*, *Spirochaetes* and *Chrysiogenetes* under micro-aerobic conditions may also carry out Hg-methylation [67]. Arsenic methylation is carried out by microbes containing the enzyme adenosylmethionine methyltransferase (ArsM), which convert As(III) to monomethyl-arsonous acid [14,18]. Subsequent methylation may change it into dimethyl-arsinous acid and trimethylarsine [68]. Some fungal species e.g., *Acidomyces acidophilus*, *Aspergillus niger* and *Penicillium expansum* have shown higher efficiency to convert inorganic As to highly methylated forms like trimethylarsine [69]. However, bacterial species (e.g., *Arsenicibacter rosenii*; species from Phylum Proteobacteriota and species from genus *Clostridium*) and algal species (*Chlorella vulgaris* and *Nannochloropsis* sp.) produce mono- and di-methylated arsenicals [70,71,72,73].

Demethylation is the enzymatic reversal process, converting toxic organometallics (MeHg) back to less bioavailable inorganic forms Hg^2+^. Demethylation is mediated by the microbes through reductive and oxidative pathways. Reductive demethylation is catalyzed by mercury resistance (mer) system which breaks the carbon–Hg bond and subsequently convert inorganic Hg^2+^ to elemental Hg^0^ via mercuric reductase (merA) enzyme at higher Hg^2+^ concentrations [74]. However, the mer-independent oxidative demethylation carried out by certain strains of anaerobic bacteria and aerobic methanotrophs at relatively low Hg^2+^ concentrations, converting methyl Hg to Hg^2+^, CO_2_, and CH_4_. On the other hand, demethylation of arsenicals is done by some bacterial species such as *Bacillus* sp. MD1 [26], *Citrobacter* sp. G-C1 [75], and algal *Nostoc* sp. PCC 7120 [76] through ArsI enzyme that catalyzes demethylation of As(III)-methylated species.

Across diverse microbial species, the heavy metal detoxification mechanism is dominated by biosorption, bioaccumulation, and redox-driven metabolic pathways. Cell wall functional groups and EPS results in metal sorption capacities determined by (*Q_max_*) often exceeding 100 mg/g in some *Bacillus*, *Enterobacter*, *Klebsiella*, *Lysinibacillus*, *Pseudomonas*, *and Rossellomorea* strains, particularly for Cd^2+^, Pb^2+^, Cu^2+^, Ni and Cr species (Table 1). As summarized in Table 1, that biosorption on cell surface and EPS-rich systems consistently showed higher metal removal compared to bioaccumulation. For redox-sensitive contaminants such as Cr(VI), Hg^2+^, and As(III), enzymatic reduction or oxidation mediated by mer operons, chromate reductases, and As(III)-oxidases contributed to metal detoxification. Collectively, analyses indicate that surface-mediated sorption dominates initial metal removal, whereas metabolic and enzymatic processes govern detoxification, stabilization, and long-term remediation. These findings emphasize the importance of integrating microbial physiology, surface chemistry, and operational configuration when designing microbial remediation strategies.

## 4. Nutrient Regulation of Microbial Metabolism and Community Structure

Microbial remediation efficiency is highly dependent on the availability and stoichiometry of essential nutrients, particularly carbon, nitrogen, phosphorus, and sulfur. These elements act as basic skeleton of the cellular constituents and functional macro-molecules which regulate the energy and biosynthetic pathways that control various metabolic processes including heavy metal detoxification.

### 4.1. Carbon Source Regulation: Energy Supply and Microbial Activity

The type and availability of the carbon source is a primary determinant of microbial growth rate and metabolic activity, which in turn affect their detoxification efficiency. Microbial mechanisms like bioaccumulation, reduction, and efflux require bioavailable carbon source (glucose, acetate) for the generation of ATP and NAD(P)H for energy and reduction process. The heterotrophic bacterial species such as *Escherichia coli*, *Bacillus subtilis*, *Lactococcus lactis*, and *Vibrio cholerae* use D-glucose as preferred carbon source over the other saccharides. In the presence of multiple carbon sources, bacterial growth exhibits a diauxic shift also termed as carbon catabolite repression (CCR) [113]. A condition where less-preferred saccharide (e.g., lactose) is available alongside glucose, the genes required to metabolize the alternative carbon source (like the lac operon in case of lactic acid) are not expressed [114]. Similarly, in many fungal species, including *Saccharomyces cerevisiae* and many filamentous fungi such as *Aspergillus* and *Penicillium*, glucose serves as the preferred carbon source and results in the fastest growth rate. In the presence of plenty of glucose, zinc-finger transcriptional repressor Mig1 binds to the promoter regions of the alternative carbon source metabolism genes (e.g., *GAL1*, *GAL7*, *GAL10*) and effectively shut down their transcription [115]. Heterotrophic algal species exhibit considerable flexibility in carbon-source utilization. Facultative heterotrophs species like *Chlorella vulgaris and Scenedesmus obliquus* prefer glucose [116] while species, such as *Euglena gracilis,* utilize ethanol as carbon source [117]. There are also exceptions in carbon-source preference among different microorganisms such as fungi, which may also preferentially utilize other soluble sugars including fructose, mannose, maltose and sucrose [118]. Similarly, *Pseudomonas putida* prefers succinate and fructose over other types of the sugars [119].

In natural environments, CCR critically affects the metabolism of diverse carbon sources such as cellulose, hemicellulose, lignin, and chitin and can influence the decomposition of organic residues and their residence time [120,121]. Many bacterial species (*B. subtilis*, *B. pumilus*, *P. fluorescens*, *P. putida*, *and Clostridium species)* secrete extracellular hydrolytic enzymes, including proteases, amylases, lipases, and peroxidases, to degrade complex organic molecules to utilizable carbon sources [122]. However, under anaerobic condition, species like *Clostridium* use a multi-enzyme complex called cellulosome containing hydrolytic enzymes (glycoside hydrolases) to enhance cellulose degradation efficiency by increasing enzyme and substrate interaction [123]. Compared with bacteria, fungi possess the ability to degrade complex organic molecules by producing extracellular enzymes such as exoglucanases, endoglucanases, β-glucosidases, xylanases, mannanases, laccases, manganese peroxidases, and lignin peroxidases that enable them to grow and multiply under complex organic food supply [124].

Carbon limitation often inhibits growth but can also stimulate the overproduction of extracellular polymeric substances (EPSs). EPSs contain abundant functional groups that significantly enhance the biosorption, acting as a defense and sequestration barrier [125]. On the other hand, CCR in rhizosphere suppresses certain microbial processes such as siderophore production, organic acid secretion, and metal-transforming enzyme activities needed for microbial metal immobilization and detoxification. Consequently, the availability and plant uptake of toxic metals like Cd^2+^, Pb^2+^, and As(III) increase. CCR promotes respiration, causing localized reduced conditions and transforms the metals into more mobile and toxic forms, i.e., As(III), CrO_4_^2−^ and Cr_2_O_7_^2−^ ions.

### 4.2. Nitrogen and Phosphorus Regulation: Key to Cell Synthesis and Enzyme Activity

The kinetic efficiency of microbes is governed not only by the availability of carbon sources but also by the optimal supply of other nutrients required to maintain the stoichiometric C:N:P ratio of microbial biomass (e.g., 60:7:1 for most soil microbes), which is critically important [126]. This ensures that the energy derived from carbon source is optimally utilized for the synthesis of biological macro-molecules as well as complex enzymes required for various metabolic processes [127]. Imbalance in the C:N:P ratio changes the soil microbial community structure. High C:N ratio (e.g., above 30:1 in compost) favors fungal growth over bacteria because fungi naturally possess a higher median C:N ratio (around 11:1 or higher), whereas bacteria are more N-rich, typically exhibiting C:N ratio of 3:1 to 10:1 [128,129]. Effects of C:N ratio on microbial community structure in forest soil demonstrated that the fungal biomass N and P biomarkers were significantly and positively related to soil C:N ratio, while bacterial biomarkers were negatively related, confirming this competitive advantage [130]. Furthermore, growth media with a low N:P ratio shift the community structure towards N-fixing cyanobacteria outcompeting the non-N-fixing algal species [131]. In addition, nutrient ratios also influence distribution of heavy metal resistance genes by favoring microbial populations capable of simultaneously maintaining nutrient cycling and metal detoxification processes. Microbes such as *Bradyrhizobium*, *Nitrospira*, *Steroidobacter*, and *Luteitalea* might play important roles in regulating C, N, P cycling and heavy metal resistance [132].

Limited N availability restricts microbial growth and enzyme synthesis including chromate reductase required for Cr(VI) detoxification. It also diminishes availability of N-containing cofactors, like NADPH, required to neutralize ROS [133]. In addition, N is part of the flavins (FAD and FMN) containing coenzymes which take part in redox reactions. It is also part of DNA and RNA needed for cell reproduction and expression of genetic traits responsible for metal transformation. The availability and chemical form of N (e.g., NH_4_^+^, NO_3_^−^, or organic N from peptone) dictate growth rates of detoxifying microbes. Therefore, the type of N fertilizer and its application rate is crucial for optimizing the performance of bioremediation systems.

Availability of P regulates microbial community structure and functional potential of heavy metal detoxifying microbes through microbial biomass production by supporting ATP production, nucleic acid synthesis, and phospholipid formation. In addition, soil P availability affects the abundance of phosphate-solubilizing microorganisms and metal-tolerant taxa that can produce substances like siderophores, organic acids and EPS for metal-complexation and biosorption [134,135]. The formation of insoluble metal–phosphate complexes by phosphate ions can also prevent the bioavailability and toxicity of metals such as Pb^2+^, Cd^2+^, and Zn^2+^ [40]. Excessive P inputs can interfere with microbial processes and change their communities [136]. Therefore, optimized and site-specific P management is required to shape microbial community composition and enrich populations capable of heavy metal transformation through enzymatic reduction, precipitation and sequestration processes.

### 4.3. Sulfur Regulation: Involvement in Sulfate Reduction and Heavy Metal Precipitation

Sulfur transformations in the soil carried by soil microbes include reduction, oxidation, and assimilation. Sulfate-reducing bacteria (SRB) use SO_4_^2−^ as a terminal electron acceptor, producing hydrogen sulfide that precipitate with heavy metals as insoluble metal sulfides [137]. Conversely, heterotrophic microbes use sulfur through assimilatory sulfate reduction (ASR) to incorporate it into essential amino acids like cysteine and methionine [137,138], while sulfur-oxidizing bacteria convert reduced sulfur compounds back to sulfate, enhancing sulfur bioavailability for plants and microbes, which improves soil fertility and crop yield [47].

Beyond assimilation, sulfate reduction by microbes has significant environmental consequences. The sulfide produced by the reduction of sulfate precipitates heavy metal cations as insoluble metal sulfides and effectively removes (>90%) Cd^2+^, Pb^2+^, Ni^2+^, Fe^2+^, Mn^2+^, Cu^2+^ and Cr(III) [139,140]. During the precipitation process, the microbial biomass serves as a nucleation site for metal precipitation [139]. The stability of metal–sulfide complexes is explained by the solubility product constant (*Ksp*) and metals with a lower *Ksp* precipitate preferentially. The general order of precipitation with sulfide follows as Cu^2+^ > Pb^2+^ > Cd^2+^ > Zn^2+^ > Ni^2+^ > Fe^2+^ > Mn^2+^ [141,142]. In addition, sulfate reduction increases the medium pH that further enhances metal precipitation and detoxification [143]. The metal precipitation and removal efficiency is influenced by microbial community structure, type and concentration of the metal/s, carbon source availability, pH, and other environmental factors [140].

The data presented in Table 2 demonstrate that SRB, especially *Desulfovibrio*-dominated consortia, exhibited high metal removal efficiencies (>90–100%) including Cd^2+^, Pb^2+^, Zn^2+^, Cu^2+^, Ni^2+^, Fe^2+^, Mn^2+^, and As(III). Mixed SRB consortia outperform single strains, due to synergistic metabolic activity, increased functional resilience, and metal–sulfide precipitation, achieving effective remediation even at higher metal concentrations (e.g., Ni^2+^ > 400 mg/L and Fe^2+^ > 800 mg/L). Optimal metal remediation efficiency has been mostly observed between pH 6.5 and 8.0, predominantly through metal–sulfide precipitation, redox-driven metal speciation changes, and biosorption in multi-metal-contaminated systems.

### 4.4. Case Study Analysis: Organic Amendment Enhancing Microbial Chromium Reduction

Trivalent chromium Cr(III) in traces is essential for living beings while Cr(VI) is highly toxic, mobile and carcinogenic. Therefore, reduction of Cr(VI) to less toxic, less mobile Cr(III) is a key bioremediation strategy. Application of organic amendments such as humic and fulvic acids and simple organic acids (e.g., tartrate, malate, lactate, and pyruvate) facilitate Cr(VI) reduction through electron transfer by microbes [153]. Additionally, hydroxyl, and carboxyl groups of organic residues directly reduce Cr(VI) to Cr(III) under low pH and high organic carbon content. The resultant Cr(III) cation can be easily immobilized through adsorption and precipitation compared to Cr(VI) at prevails as the chromate anion (CrO_4_^2−^ at neutral pH) and dichromate anion (Cr_2_O_7_^2−^ at lower pH).

Certain bacterial species, such as *Bacillus* sp. *and Pseudomonas aeruginosa*, can efficiently reduce Cr(VI) by concurrent Cr(III) immobilization on cell surfaces and/or intracellular precipitation [154,155]. In addition, SRB enhances Cr(VI) reduction largely through biogenic sulfide (S^2−^) which is a very strong reductant. Under such conditions, iron-reducing bacteria also effectively reduce Cr(VI) and facilitate Cr(III) incorporation into iron mineral lattices [156]. In contrast, fungal species often achieve Cr(VI) reduction indirectly through the secretion of powerful extracellular organic acids and reductants into their surrounding environment. However, Cr(VI) remediation using algal-bacterial systems strongly relies on organic carbon released from algae (exudates and debris) for supplying electron donors/food to Cr(VI)-reducing bacteria, thereby enhancing overall bio-reduction [157].

Biochar with abundant oxygen-containing functional groups synergistically improves Cr(VI)-reducing activity of *Shewanella oneidensis* MR-1 by promoting electron transfer and decreasing the microbe-mineral distance [158]. Modification of organic residues into their composites with inorganics significantly enhances Cr(VI) reduction with improved electron transfer, photocatalytic activity and adsorption [159]. Biochar composites with Fe or Mn-oxide also improved Cr(VI) removal by *Bacillus cereus* ZNT-03 by up to 72%, by providing electron donors and facilitating adsorption, complexation, and electrostatic attraction at the composite surfaces (Table 3).

Data compiled in Table 3 from various studies showed Cr(VI) ranges from 55.4 to 100. Consortium exhibited 95% Cr(VI) reduction efficiency compared to individual strains with a mean value of 85%. Adding various organic sources increases microbial Cr(VI) reduction and detoxification. Complex organic compounds such as molasses and yeast extract showed removal efficiency within the range of 98–100%, while carbon substrates such as glucose and lactate had comparatively lower efficiencies (~75–90%). Quinone-rich amendments, humic substances and dissolved organic matter resulted in higher Cr(VI) reduction efficiency probably due to their function as electron shuttles, enabling indirect Cr(VI) reduction. Integration of biotic and abiotic systems, such as biochar–FeS_2_ composites or sludge-derived carbon materials, showed higher effectiveness (96% Cr(VI) reduction efficiency) due to synergistic coupling of microbial metabolism with mineral-mediated redox reactions and surface precipitation. The addition of redox-active materials achieved reduction within 24 h, while fermentation-driven systems required as much as up to 21 days to remove the contaminant. Increasing initial Cr(VI) decreased microbial reduction efficiency, dropping from 100% to as low as 68–80% at higher concentrations. Overall, the data in Table 3 highlights that carbon sources, their concentration, and electron-transfer mechanisms are the critical factors that determine Cr(VI) reduction efficiency.

## 5. Research Progress on the Application of Nutritional Regulation in Phytoremediation

### 5.1. Mechanisms of Heavy Metal Phytoremediation

Phytoremediation is a process involving the plants to remediate the heavy-metal-contaminated environments that depends on the plant’s ability to balance between plant mineral nutrients and heavy metals. Heavy metals exploit the same plant transporters used for nutrients uptake. For example, Cd is often taken up through ZIP- family or Fe^2+^ transporter IRT1, which normally mediate uptake of Zn^2+^, Fe^2+^ and Mn^2+^ [176]. Similarly, As(V) competes with phosphate (PO_4_^3−^) for uptake via phosphate transporters [177]. Fertilization of essential nutrients (Zn, Fe, P) can saturate the transporters, effectively reducing the Cd^2+^ and As(V) uptake and accumulation [178]. Nutrient such as sulfur, is a structural constituent of thiol-rich peptides responsible for intracellular heavy metal chelation and sequestration into the plant vacuoles [179]. Conversely, Ca amendments often increase soil pH and enhance precipitation of many cationic heavy metals like Pb^2+^ and Cr(III), thereby restricting their uptake by the plant roots [180]. Since phytoextraction efficiency is proportional to the harvestable biomass, optimal N and P and other essential mineral element fertilization is necessary to support the high growth rates required to maximize total metal removal from the soil. In short, nutrient management directly affects the metal acquisition, detoxification, and immobilization in plants; henceforth, optimizing the nutrient interaction can help to develop a cost-effective phytoremediation technology.

Phytoextraction requires plants to absorb, uptake, translocate, and sequester heavy metals into harvestable plant parts. Plants with phytoextraction potential generally belong to families including *Asteraceae*, *Brassicaceae*, *Buxaceae*, *Cunoniaceae*, *Euphorbiaceae*, *Flacourtiaceae*, *Phyllanthaceae*, *Rubiaceae*, *Salicaceae*, and *Violaceae*. To date, more than 700 metal-hyperaccumulating species belonging to 52 families and approximately 130 genera have been reported [181]. Among these, the *Brassicaceae* with species like *Noccaea caerulescens* showed exceptional multi-metal hyperaccumulation, surpassing thresholds for both Zn and Cd with concentrations of up to 56,200 µg/g DW and 3410 µg/g, respectively [181,182]. Similarly, *Hirschfeldia incana* from the same family accumulated Pb to over 30,000 mg/kg DW in its shoots, demonstrating strong Pb hyperaccumulation capacity. This plant metal hyperaccumulation has been associated with higher expression of metal transporter *HiHMA4* and *HiMT2a,* and metal sequestration metallothionein genes [183]. *Alyssum murale* in this family showed high Ni accumulation of up to 25,400 µg/g DW through its binding with organic ligands such as malate and histidine [184]. Additionally, *Alyssum murale* accumulated cobalt > 10,000 µg/g DW by forming cobalt-rich mineral precipitate(s) in leaves tips and margins [185]. Tropical Ni hyper accumulators from the Phyllanthaceae and Rubiaceae families such as *Psychotria* species and *Phyllanthus balgooyi* can accumulate exceptional high Ni concentrations and sometimes its concentration may reach over 16 wt% Ni in their phloem sap and epidermal cells [186,187] (Table 4). Certain genera such as *Pancheria* and *Homalium* from the families *Cunoniaceae* and *Flacourtiaceae* are also considered to be Ni hyperaccumulators. A fern species such as *Pteris vittata* is another classic example of a hyperaccumulator that may accumulate As in its fronds up to 2.7% of DW [188]. Some plant species owing to their rapid growth such as members of *Salicaceae* (*Salix* spp.) and *Brassicaceae* (e.g., *Brassica juncea*) may not accumulate extremely high concentrations of heavy metals, but their rapid growth and substantial biomass production make them highly effective for the phytoextraction of the heavy metals.

In contrast to hyperaccumulation, phytostabilization immobilizes heavy metals to mitigate their environmental risks, relying on plants’ ability to tolerate high concentrations of metals and effectively sequester them at the root surface or in the rooting medium. Stabilization process includes physical stabilization, where dense fibrous root systems (e.g., in *Poaceae* like *Vetiveria zizanioides*) reduce soil erosion, while deep-rooted woody plants (e.g., *Salicaceae* like *Salix* spp.) reduce metal leaching with water percolation [212]. *Salix viminalis* and *Salix purpurea* showed deposition of As and Pb mainly in roots with a limited translocation to shoots, indicating strong pphytostabilizationpotential [213]. The stabilization potential of these species is further enhanced with the soil amendments like biochar [214]. On the other hand, biochemical mechanisms involving complexation and chelation of heavy metals by plant root exudates, cell wall constituents, and intracellular chelation by phytochelatins and metallothioneins, play a key role in phphytostabilizationThese traits are widely expressed by many tolerant species across families like *Asteraceae* (e.g., *Helianthus annuus*) and *Brassicaceae*. Negatively charged organic macro-molecules, e.g., pectin, hemicelluloses, and proteins, adsorb heavy metal and limit their mobility and toxicity [212]. Furthermore, plants from *Fabaceae* family contribute to phyphytostabilizationrough N fixation, which promotes the establishment of a stable and dense plant cover, thereby reducing the overall mobility of heavy metals like Pb^2+^, Cd^2+^, and Zn^2+^. The combined effect of these microbial and biochemical processes ensures ecological safety at contaminated sites by maintaining low metal mobility in contaminated soils.

Phytovolatilization involves the biotransformation of the metal/metalloid and its conversion to volatile form. The mechanism is highly specific to some metal/metalloid such as selenium (Se), Hg and As. For instance, Se is taken up through SO_4_^2−^ transporters, converted to seleno-amino acids (seleno-cysteine and seleno-methionine), and subsequently methylated by enzymes into dimethyl selenide which are then released to the atmosphere [213]. Conversely, Hg phytovolatilization typically relies on the expression of the bacterial mer operon genes in plants that has been successfully introduced into tobacco [215]. During this detoxification process, an enzyme MerB (organomercurial lyase) converts methyl Hg into Hg^2+^. Subsequently, an enzyme MerA reductase reduces Hg^2+^ in an NADPH-dependent reaction to a gaseous elemental Hg^0^ [216]. This combined MerA/MerB strategy has been introduced into rice, minimizing Hg accumulation and build-up in the food chain [217]. Unlike Hg and Se, As phytovolatilization is a complex and less studied mechanism with a limited role in As detoxifications in plants as minimal amount of As can be methylated into volatile compounds such as arsines [218]. This process is inefficient and often relies on associated microorganisms rather than just on plant enzymes. Therefore, greater focus is on the use of As hyperaccumulators, i.e., *Pteris vittata*, along with other species, in combination with plant-growth-promoting rhizobacteria (PGPR), and nutrient management strategies have been proposed to enhance overall phytoremediation efficiency.

### 5.2. Nutrient Regulation to Enhance Phytoremediation Efficiency

Nitrogen fertilization is strategically used to enhance phytoextraction, primarily for mobile metals like Cd [192,193]. Metal-enhanced phytoextraction efficiency is associated with NH_4_^+^-N, which enhances Cd^2+^ uptake and translocation by reducing soil pH [35,202]. NH_4_^+^ supply significantly increased Cd accumulation by 44.9% and 31.1% in Populus clone Nanlin 1388 and Nanlin 895, respectively, compared to NO_3_^−^ treatment [219]. Similarly, Cheng et al. [220] reported increased Cd accumulation in *Solanum nigrum* L. (23%) and *Carpobrotus rossii* (20%) under NH_4_^+^ than under NO_3_^−^ supply. Nitrogen application in *Solanum nigrum* L. not only increased Cd phytoextraction (111.8 to 523.5%) but also substantially increased plant shoot biomass (113.7 to 482.2%) [221]. Similarly, application of urea in *Lolium multifolorum* significantly increased Cd accumulation by 85% and shoot biomass by 213% [222]. In addition, *Nicotiana tabacum* L. supplied with NH_4_NO_3_ significantly increased Cd (93%) and Pb (129%) accumulations along with the aboveground biomass (128%) [223]. In contrast, some studies also reported that NO_3_^−^ application can enhance Cd accumulation in sweet sorghum [224]. Ammonium nitrate fertilization (60 kg/ha) promoted the accumulation of Pb (44%), Cd (140%), and Zn (442%) in leaves of *Silybum marianum* [225]. Additionally, N enhances the Transfer Coefficient (TC) with improved metal root-to-shoot translocation, needed for effective phytoextraction [226].

Phosphorus fertilizer application is primarily used for metal phytostabilization as P containing compounds (like hydroxyapatite or diammonium phosphate) convert bioavailable metal in insoluble minerals, such as Pb^2+^ which forms highly stable and insoluble mineral pyromorphite (Pb_5_(PO_4_)_3_X) [227]. Studies confirm that P fertilization was highly effective in immobilizing Pb, significantly reducing its water solubility by 72–100%, and phyto availability by 15–86% [228]. Furthermore, the application of hydroxy-apatite-modified biochar mitigates Pb^2+^ leaching risks by effectively immobilizing it in the soil [229]. Additionally, potassium (K) plays a vital role in maintaining plant physiological processes and stress tolerance to sustain higher biomass needed for efficient remediation. The element is important in the regulation of the uptake channels/pumps that might otherwise be saturated by heavy metal cations [230].

Calcium plays vital role in stress signaling and maintaining the integrity of the cell wall and its membrane, which promotes root growth and uptake of nutrients under contaminated conditions. It also competes with Cd^2+^, reducing its uptake by ~31% in *Brassica juncea* [231] and alleviating metal toxicity and enhancing plant biomass. Calcium deficiency in *Morus alba* restricted root tip growth, while its adequate supply ensured an extensive root system, enhancing contaminant interception and detoxification [232]. Magnesium as the central atom of the chlorophyll molecules directly contributes to the synthesis of photosynthates required for shoot and root growth. Additionally, it plays an important role in enzyme activation and sugar translocation, ensuring that the necessary nutrients are transported effectively to the actively growing root zone [233]. Application of magnesium to *Torreya grandis* improved shoot growth by 12.0%, while it reduced Pb content by 32.5% compared to sole Pb treatment [234]. Similarly, magnesium in *Brassica rapa* L. improved shoot dry biomass by ~120%%, while it reduced Cd concentration by 40% [235]. Foliar application of nano magnesium-oxide in *Mentha arvensis* reduced Cd accumulation by 30.6%, and increased its biomass by 14.7% by improving antioxidative activity [236]. In *Arabidopsis*, magnesium mitigated Cd toxicity by maintaining plant Fe homeostasis hence protecting photosynthetic apparatus from damage [237].

Sulfur (S) is pivotal in internal detoxification that helps the plants to survive and effectively sequester metals. It serves as a precursor of S-containing compounds like glutathione (GSH) and phytochelatins (PCs) [179]. These compounds serve as plants’ primary defense, acting as chelating agents that bind, detoxify and sequester metals like Cd and As into the vacuoles [238]. The internal detoxification of Cd can be enhanced by supplying optimal S which can contribute to a total accumulation of Cd in plant tissues [239]. Thus, S application does not necessarily prevent metal uptake but it improves the metal tolerance and hyperaccumulation, thereby enhancing phytoextraction efficiency [240].

Micro-nutrients, like Zn, Fe, Mn, and Cu, mitigate heavy metal toxicity by competing with toxic heavy metals for uptake sites and improving overall plant growth. For example, Zn application reduced Cd accumulation in shoots of *Sedum plumbizincicola* by 20–40% compared to Zn-deficient soil [241]. The competition for uptake sites reduced the concentration of Cd in shoot, thereby improving plant health and overall biomass [242]. The application of Fe and Mn supported chlorophyll biosynthesis and enhanced antioxidant defense. As a result, root elongation increased, thereby facilitating contact with heavy metals [243]. The addition of Cu to *Pteris vittata* increased the accumulation of As by 1.4-folds and its biomass by 1.2-folds relative to sole application of As at the rate of 50 µM [244]. Cadmium in the growth medium inhibits Mn uptake; however, Mn fertilization improves nutrient equilibrium such as improved NO_3_^−^ uptake by maize [245]. In essence, targeted nutrient supply ensures plant survival and promotes high biomass for effective contaminant removal.

### 5.3. Case Study Analysis

#### 5.3.1. Synergistic Effect of Metal Chelators on Heavy Metal Phytoextraction

Metal chelators enhance phytoextraction due to their ability to form metal–chelate complexes increasing plant metal uptake [246]. Higher phytoremediation efficiency is achieved with the increased bioavailability of heavy metals in the soil. Most heavy metals such as Pb, Cd, and Zn, are typically immobile in the soil, bound to soil particles and unavailable for plant uptake. Meta chelators strongly bind to polyvalent heavy metal ions, such as Pb^2+^, Cd^2+^, Zn^2+^, and Ni^2+^, forming highly stable metal–chelate complexes [75]. This chemical reaction desorbs the metal from the soil and increases its concentration in the soil solution. Crucially, plants can rapidly absorb and efficiently translocate metals through pathways that allow for bypassing typical selective uptake mechanisms from the roots to the shoots [247]. In several cases (*Brassica juncea*, *Zea mays*, and *Panicum virgatum*), EDTA increased shoot metal concentrations by >30–40-fold, especially under highly contaminated soils [248], Table 5. However, the high mobility and limited biodegradability of EDTA–metal complexes pose a significant environmental risk, primarily the leaching of the heavy metals into groundwater, which is a major concern for large-scale applications [248]. In addition, EDTA effectiveness is highly dose-dependent and may not match with plants’ uptake system. Biodegradable synthetic chelators such as GLDA, MGDA, EDDS, NTA and IDSA have demonstrated moderate effects on plants’ metal uptake enhancement (1.3–10×). However, these chelators have shown comparable results to the EDTA in *Zea mays* and *Sedum hybridum* for Cd and Zn uptake with less environmental persistence and toxicity, making them the promising alternatives to EDTA [249,250], Table 5. Chelator effectiveness varies with plant species, metal type, soil conditions, and dosage, requiring careful optimization to balance metal uptake and plant health [251].

The data presented in Table 6 clearly show the positive effects of chelators on the phytoextraction of Cd and Pb, followed by Zn, Ni, and Cu. Under a multi-metal contamination system, these chelators enhanced the uptake of all metals; however, uptake varied depending on the metal. The effectiveness of chelators in inducing phytoextraction was limited in the case of Cr and uranium (U). Various *Brassicaceae* species (*Brassica rapa* and *B. napus)* along with fast-growing grasses (*Panicum virgatum* and *Stipa arabica*) and leafy plants (*Solanum nigrum* and *Portulaca oleracea*) consistently exhibited a significant increase in metal accumulation in response to chelator application, confirming their suitability for induced phytoextraction (Table 5). However, woody and ornamental plant species had more variable responses, highlighting species-specific physiological constraints. Overall, high variability in the enhancement factors across studies is attributed to differences in plant species, type of the heavy metal, pH, growing conditions, type of the chelator and the applied chelator dose [251,268]. Overall, using biodegradable chelator offers a more sustainable approach to enhancing phytoextraction of heavy metals from contaminated soils.

#### 5.3.2. Nitrogen Fertilization Enhances Arsenic Uptake by *Pteris vittata*

Arsenic is a potent Group 1 carcinogen, and its contamination, especially in agricultural soil through natural and anthropogenic sources poses a major global risk, affecting over 230 million people worldwide [269]. The natural background of As in soil ranges from 0.2 to 40 mg/kg [270] and remediation action in farmland often starts around 40 to 60 mg/kg (varies by region). Phytoremediation using *Pteris vittata* is the most cost-effective for soils with As concentrations typically ranging from 20 mg/kg to 200 mg/kg [17,271]. *Pteris vittata* is an important As hyperaccumulator plant, primarily taking up As(V) through phosphate transporters (PvPht1;3 and PvPht1;4), subsequently reducing it by As(V)-reductase PvACR2 or glutaredoxin PvGRX5 to As(III) before its translocation to fronds [272]. The translocation of As(III) to fronds is differentially regulated depending on the As supply from the root. Under limited As supply, the As(III) is mainly accumulated in young fronds, while at higher concentrations the deposition is shifted to mature fronds. This efficient and well-coordinated mechanism allows *Pteris vittata* to accumulate over 27,000 mg/kg of As in its dry biomass [273], positioning it as a model species for phytoextraction and sustainable, green alternative to conventional soil cleanup methods. Various *Pteris vittata* ecotypes exhibit distinct strategies depending on the As concentration of their native habitat. Populations from low-As ecological zones showed higher concentration efficiency, whereas ecotypes from high-As zones were more tolerant and exhibited higher biomass accumulations, underscoring the importance to match the site-specific genotype selection to the cleanup of contaminated site [274].

It has been reported that *Pteris vittata* distribution is strongly correlated with climatic factors, and its phytoremediation potential is not uniform, but varies among ecotypes and is also influenced by nutrient management [275,276]. For instance, N fertilization generally leads to higher fern biomass, and therefore, higher total As accumulation. The plants with additional N supply accumulate approximately 2.3% more As compared to the control [277]. Data presented in Table 6 shows that NH_4_^+^-N fertilizers (e.g., NH_4_HCO_3_, (NH_4_)_2_SO_4_, NH_4_Cl, MAP, and DAP) generally resulted in higher As accumulation in fronds and enhanced translocation factors (TFs) compared to the NO_3_^−^-N nutrition. This trend is evident in the Chenzhou ecotype, where NH_4_-N resulted in As ranges from 6309 to 7501 mg/kg DW and TF values of 12–16, higher than those observed under NO_3_^−^ supply. Similar enhancement of As accumulation and shoot biomass under NH_4_-N fertilization was reported for Indian and Chinese ecotypes, particularly with NH_4_-phosphate fertilizers.

**Table 6:** Application of Nitrogen form on Arsenic uptake and translocation (determined Translocation Factors; TF from root to fronds) by hyperaccumulator *Pteris vittata.*

Ecotype Source	Dominant N Form	As Conc. (Fronds, mg/kg DW)	TF (Frond/Root)	Citation
*Pteris vittata* (Chenzhou, China)	NH_4_HCO_3_; (NH_4_)_2_SO_4_ (NH_4_^+^ source)	6309–7501	12–16	[277]
	(KNO_3_; Ca(NO_3_)_2_- NO_3_ source	4973–5463	9.9–14.9	
	Urea	5597	11.6	
*Pteris vittata* (Milestone Agriculture, Inc., FL)	Control	124	1.76	[278]
	NH_4_^+^ (NH_4_Cl)	130	3.63	
	NO_3_^−^ (NaNO_3_)	47.3	0.37	
*Pteris vittata* (Nadia district, West Bengal, India)	Control	2254	9.49	[275]
	SSP (single super phosphate)	3159	9.25	
	DAP (di-ammonium phosphate) NH_4_^+^ source	3803	10.28	
*Pteris vittata* (field-grown Mediterranean climate, CA, USA)	Control (background soil N)	~1260 (mean across various soil textures)	≈20–≈28	[276]
	(NH_4_)_2_SO_4_	≈498	≈10–≈24	
*Pteris vittata* (Shimen County, Hunan Province, China)	Control	1150–1200	≈26.1	[279]
	CSP (calcium super phosphate)	730–780	≈25.9	
	MAP (mono-ammonium phosphate) NH_4_ source	820–880	≈17.4	

Note: “≈” Represents estimated value from published figures using visual digitization.

In contrast to NH4^+^, NO_3_^−^ nutrition (e.g., KNO_3_, Ca(NO_3_)_2_, NaNO_3_) decreased As concentrations in frond as well as TFs, as clearly shown in the Florida ecotype. Field-based studies further indicate that background soil N and NH_4_-sulfate fertilization can substantially decrease As accumulation relative to controls, highlighting the influence of soil chemistry and N-induced changes in rhizosphere pH, phosphate availability, and As(V)/PO_4_^3−^ competition. Overall, the data shows that NH_4_^+^ nutrition encourages As phytoaccumulation in *P. vittata* by increasing As uptake, root-to-shoot translocation, and in several cases, shoot biomass. However, plant responses to N nutrition are ecotype-specific and field-condition-dependent, requiring the need for site- and genotype-based N management strategies in As phytoremediation.

## 6. Nutritional Synergy in Microbe–Plant Heavy Metal Remediation

### 6.1. Microbes, Plant Nutrients and Heavy Metal Interaction in the Rhizosphere

Microbes in the rhizosphere, particularly plant-growth-promoting rhizobacteria (PGPR), employ several complex and synergistic processes to increase plant nutrient availability and uptake. PGPR effects on plant growth can be direct or indirect. As recently reviewed, a comprehensive understanding of root exudate-driven microbial recruitment and the resulting feedback loops are fundamental to engineering the rhizosphere for both nutrient acquisition and stress mitigation [280]. PGPR significantly increase the bioavailable nutrients in the soil by solubilizing minerals, for instance P solubilization by the microbes. Microorganisms secrete organic acids (e.g., gluconic, lactic, and 2-ketogluconic acid). These organic acids solubilize the tricalcium phosphate or aluminum/iron phosphates, releasing the plant-available orthophosphate ions (H_2_PO_4_^−^, HPO_4_^2−^) [281]. Similarly, under Fe-limiting conditions, PGPR produce highly effective, low-molecular-weight siderophores. These chelating agents bind insoluble ferric iron (Fe^3+^) and transport the Fe–siderophore complex to the root. The siderophore-bound Fe is either taken up by the plants directly (e.g., grasses (strategy II plants) or it is reduced to Fe^2+^ before uptake in dicotyledons and non-grass monocotyledons called strategy I plants [282]. Certain mineral-solubilizing bacteria weather soil minerals containing essential nutrients like K, Zn, and Mg, making them available for plant uptake. For example, *Bacillus circulans* weathers K-rich silicate minerals like mica and feldspar releasing bound K, while the fungus *Aspergillus niger* solubilizes magnesium from talc and serpentine minerals [283]. Furthermore, microbes such as *Bacillus licheniformis* and *Penicillium* species mobilize Zn by dissolving its insoluble compounds, often simultaneously with P solubilization [284]. Microbes can also directly contribute to providing plant nutrients, primarily N, through symbiotic (*Rhizobium* in legumes) and non-symbiotic N fixation (free-living *Azotobacter*). Microbes convert inert atmospheric N_2_ into plant-useable N forms (e.g., ammonium (NH_4_^+^), amides (asparagine and glutamine in temperate legumes) and ureides (allantoin and allantoic acid in tropical legumes) [285].

PGPR synthesize and release phytohormones such as Indole-3-acetic acid (IAA) that stimulates root cell elongation and division with increased lateral branching. A larger, denser root system with mycorrhizal association provide the plant an opportunity to explore the soil zones enriched with nutrients. Microbial compounds can potentially influence the expression and activity of specific nutrient transporters, facilitating nutrient uptake from the soil solution to the root epidermis [286]. They also produce important volatile organic compounds (VOCs) that act as signaling molecules to enhance plant nutrient acquisition. For example, under S deficiency, VOCs from *Bacillus amyloliquefaciens* species promote Se and SO_4_^2−^ uptake via root nitric oxide signaling and upregulation of nutrient acquisition genes [287]. Similarly, VOCs (glyoxylic acid, 3-methyl-butanoic acid, and diethyl acetic acid) produced by *Bacillus amyloliquefaciens* GB03 induce the transcription of the Fe^3+^ reductase FRO_2_ and Fe^2+^ transporter IRT1 [288]. In addition, VOCs like N and N-dimethylhexadecylamine produced by *Arthrobacter agilis* and *Pseudomonas fluorescens*, triggers plant responses to Fe deficiency [289], while VOCs like diacetyl, released by GB03, enhances plant’s responses to P deficiency, by inducing salicylic acid (SA)- and jasmonic acid (JA)-mediated signaling pathways [290]. Together, these actions transform the rhizosphere into a highly active nutritional zone, a key prerequisite for effective phytoremediation. Efficacy of phytoremediation largely depends on the bioavailability of metals to the plants and the toxicity of metal on selected plant species [291]. To overcome these limitations, inclusion of PGPR with nutritional regulation can provide synergistic effects within combined remediation systems, thereby maximizing plant health, microbial diversity and contaminant removal [292]. Exogenous mineral nutrient supply mitigates heavy metal toxicity by changing the nutrient balance, reducing metal uptake and translocation, and improving antioxidant system making plants better adapted for effective phytoremediation. Some PGPR, especially from the genera *Bacillus*, *Pseudomonas*, *and Azotobacter*, are better adopted and continue to function efficiently for P solubilization and N fixation under heavy metal stress [293]. *Bacillus siamensis* R27 solubilized P at 385.11 mg/L despite Cd presence [294]. Chromium-tolerant *Azospirillum brasilense* EMCC1454 showed P solubilization potential in the presence of 260 µM K_2_Cr_2_O_7_ [295]. Similarly, P solubilization activity was observed by copper-resistant *Pseudomonas* strains in maize and sunflower [296]. Simultaneously, it has been reported that symbiotic association of *Anthyllis vulneraria* with *Mesorhizobium metallidurans* fixed up to 80% of total plant N under highly Zn-, Pb-, and Cd-contaminated conditions [297]. In addition, *Azotobacter chroococcum* strain CAZ3 and *Azospirillum brasilense* EMCC1454 maintained N fixation under metal stress of Pb (2000 µg/mL) and Cr (260 µM) and improved plant growth [295,298]. Wheat inoculation with *Pseudomonas putida* Khsr4 increased uptake of K, Ca, and Mg and reduced Cr uptake by lowering soil pH [299]. Similarly, *Pseudomonas* strains resistant to Cu increased K, calcium, and magnesium accumulation in maize and sunflower by ACC deaminase with reduced ethylene level [296].

Beyond macronutrients, PGPR enhanced micronutrient (Fe, Zn, Mn, and Cu) availability by secreting siderophores which have dual effect on mobility of Fe as well as on other divalent metal cations, such as Cd^2+^, Pb^2+^, and Zn^2+^ [300]. This chelation action enhances the mobilization and bioavailability of the heavy metals for phytoextraction and/or local metal sequestration in the rhizosphere [301]. The VOC and N,N-dimethylhexadecyl-amine, produced by *Arthrobacter agilis,* trigger systemic Fe deficiency response leading to the upregulation of Fe transporters (e.g., FRO2 and IRT1) that facilitate the uptake of both Fe and other heavy metals [302].

Microbial diversity and key functions, such as nutrient cycling, are affected by heavy metal stress. Heavy metal stress significantly reduces microbial diversity often leading to a loss of key ecological functions such as biogeochemical cycling of the nutrients derived from the organic residues. Nutrient addition to the heavy-metal-contaminated soils acts as a selective pressure, favoring the metal-tolerant microbes. Heavy metals like Pb, Cd, Zn, and Se inhibit sensitive microbial groups (e.g., Acidobacteria, Chloroflexi, and Archaea) while favoring metal-tolerant taxa such as *Proteobacteria*, *Actinobacteria*, *and Firmicutes*, resulting in altered community composition and reduced richness [303]. For instance, sulfate-reducing bacteria (*Desulfovibrio vulgaris),* supplied with emulsified corn oil as carbon source, resulted in increased Cd^2+^, Mn^2+^, and Cu^2+^ sulfide precipitation [148]. Soil inoculation with ureolytic bacteria induced calcite precipitation that immobilized Pb^2+^, Zn^2+^, and Mn^2+^, improving heavy metal bioremediation [304]. In the soil co-contaminated with metals like Cd or Pb, spore forming Bacillus species (*Bacillus subtilis*, *Bacillus cereus*, and *Bacillus thuringiensis*) rapidly establish dominance while their less-tolerant competitors remain suppressed [305]. Organic amendments support *Pseudomonas* and *Bacillus* with Cr(VI) reductase, and favors the conversion of toxic Cr(VI) to Cr(III) while increasing their abundance and function in the rhizosphere [306]. In addition, nutrient amendment boosts microbes (e.g., *Stenotrophomonas* and *Pseudarthrobacter)* that mitigate heavy metal-induced ethylene stress in plants by ACC deaminase activity [307]. Similarly, nutrient availability supports the PGPR ability to produce high levels of antioxidants and osmolytes (e.g., proline), which protect the plant against heavy metal-induced oxidative damage, further boosting plant tolerance and biomass [180]. The composition of applied nutrients to the soil determines whether a plant adopts phytoextraction or phytostabilization. Nutrients that stimulate the microbes to produce low-molecular-weight organic acids (e.g., citric and oxalic acids) promote phytoextraction. These organic molecules acidify the rhizosphere and chelate metals, increasing their solubility and availability for plant uptake. Conversely, nutrient amendments that encourage the production of high molecular weight organics with strong metal-binding abilities result in phytostabilization and reduce metal uptake [308]. In short, the synergy between microbes and plants under heavy metal stress determines whether remediation is ecologically effective or further stresses the plants and soil.

### 6.2. Rhizodeposits and Natural Carbon Sources Driving Microbial Processes

The effectiveness of microbe-plant-combined remediation systems for heavy metal phytoextraction, relies on the consistent supply of rhizodeposits which contain a mixture of low- and high-molecular-weight compounds. It has been reported that around 20–60% of the net photosynthates are transported to the roots, while 10–20% of this amount is released as rhizodeposits [291,309]. Plant’s secreted low-molecular-weight organic compounds include sugars, amino acids, organic acids, phenols, enzymes, vitamins and some types of signaling molecules that increase microbial growth [284,291]. Organic acids, such as citric, malic, oxalic, formic, piscidic, and malonic acid from the root exudates, enhance the solubility and bioavailability of heavy metals for plant uptake [310]. In addition, root-secreted low-molecular-weight signaling molecules, such as flavonoids, support arbuscular mycorrhizal fungi spore germination, hyphal growth and root colonization [311]. The flavonoids also serve as chemo-attractants for the host-specific rhizobia and also induce nodulation (nod) genes involved in the synthesis of lipochitin oligosaccharide signaling molecules and the nod factors. Plant-secreted flavonoid-related compounds, e.g., daidzein and genistein (iso-flavonoids) and luteolin (flavone) also induce rhizobial nod gene expression with a subsequent nodule formation and N fixation [312]. Plant-produced high-molecular-weight rhizodeposits, such as mucilage (a gelatinous substance primarily composed of polysaccharides), stabilize the rhizosphere soil structure, improve water retention, and provide a physical matrix for microbial colonization. It has been reported that root mucilage from several plant species contains 78 to 97% sugars and 3 to 5% amino acids that on degradation may also serve as a source of carbohydrates for microbes [313]. In addition, cellular debris, released from root cap cells and from the exodermis, provides complex organic matter that supports a more diverse range of decomposers [314].

### 6.3. Case Study Analysis

#### 6.3.1. Effects of Arbuscular Mycorrhizal Fungi and Phosphorus on Phytoremediation

Phytoremediation effectiveness is often limited by the phytotoxicity of heavy metals and severe nutrient deficiencies, particularly P deficiency, which is common in contaminated sites. In addition, heavy metal ions cause toxicity to roots, limiting essential nutrient uptake, inhibiting plant growth necessary for effective phytoextraction [315]. To overcome these barriers, integration of P fertilization with the inoculation of arbuscular mycorrhizal fungi can provide promising results. Arbuscular mycorrhizal fungi are obligate symbionts that form extensive hyphal networks, extensively increasing the plant root surface area. The establishment of this symbiosis is mediated by specific molecular signals; for example, LysM receptor, like kinases in rice, perceive fungal lipochitooligosaccharides to promote root colonization and development [316]. However, under heavy metal contamination and P-deficient soils, both the plant and the arbuscular mycorrhizal fungi are under stress. In this case, we have reviewed how co-application of P fertilizer affects arbuscular mycorrhizal fungi function, enhancing P availability and promoting both plant growth and metal detoxification. The data compiled in Table 7 show that arbuscular mycorrhizal fungi play a critical role in heavy metal detoxification in plants. The effectiveness of arbuscular mycorrhizal fungi-induced detoxification is mainly attributed to P fertilization, heavy metal type, arbuscular mycorrhizal fungi species, and host plant. In general, heavy metals such as Cd^2+^, Pb^2+^, Zn^2+^, Al^3+^, and As negatively affect arbuscular mycorrhizal fungi colonization and hyphal development. However, some AMF species, i.e., *Rhizophagus irregularis* and *Funneliformis mosseae*, often sustain symbiosis under elevated metal concentrations. Moderate P fertilization enhances plant biomass and metal tolerance. However, high P concentrations may suppress mycorrhizal colonization. These impacts are further affected by plant genotype, particularly in systems with P metal antagonism.

Heavy metal stress decreased arbuscular mycorrhizal fungi colonization by 33.5% (Table S1), while arbuscular mycorrhizal fungi association up to 78% [325] plant growth. At the same, arbuscular mycorrhizal fungi decreased shoot metal accumulation. Phosphorus application resulted in higher plant biomass but decreased arbuscular mycorrhizal fungi colonization. Among arbuscular mycorrhizal fungi species, *Rhizophagus irregularis* exhibited maximum tolerance to heavy metal stress and retained higher colonization under metal contaminated experimental conditions. Plant symbiosis with arbuscular mycorrhizal fungi results in improved plant growth and P acquisition under metal stress, The increased plant biomass with reduced shoot metal concentrations can be due to metal dilution effect. Various mechanisms for metal detoxification, including reduced root-to-shoot translocation, retention of metals by arbuscular mycorrhizal fungi, change in of rhizosphere pH, and increased antioxidant defense observed in various studies. For As, competitive inhibition between PO_4_^3−^ and As(V) represents a dominant control pathway. Overall, the data highlights that arbuscular mycorrhizal fungi dynamically regulate metal bioavailability and induce plant tolerance by optimized P management and compatible host-fungus symbiosis. These interactions provide a sound basis for the integration of arbuscular mycorrhizal fungi-based strategies with P-fertilization for sustainable phytoremediation and metal-resistant cropping systems.

#### 6.3.2. Plant-Growth-Promoting Rhizobacteria and Organic Fertilizer Application to Remediate Heavy-Metal-Contaminated Soil

The synergistic effect of PGPR and organic fertilizer can enhance the metal bioavailability, growth and soil conditions required for phytoremediation [293]. PGPR enhance the plant’s natural phytoremediation capabilities by directly interacting with the metals and the root system [344]. The PGPR increase phytoremediation through increased metal availability by the secretion of roots exudates, phytohormones production, and inducing systemic resistance to ensure that the plant remains physiologically active and sequester the metals from the soil [345,346,347]. Under certain conditions, PGPR can also immobilize heavy metals by biosorption onto their cell walls or by precipitating them, which is useful for phytostabilization [348].

Organic fertilizers combined with PGPR provide a strong foundation for enhanced remediation. The organic matter from these fertilizers directly immobilizes heavy metals through complexation with functional groups like carboxyl and hydroxyls groups, effectively reducing the soluble and toxic metal fraction in the soil solution [349]. Furthermore, organic amendments buffer the soil pH and improve soil texture, which indirectly reduces heavy metal bioavailability and toxicity, providing a less stressful environment for plant roots and associated PGPR [350]. Crucially, the introduction of organic carbon and nutrients stimulates the growth and metabolic activity of the inoculated PGPR and native microbial communities. This enhanced microbial activity accelerates the decomposition of organic fertilizers, leading to sustained nutrient release ultimately maximizing the plant’s overall health and its capacity for sustained heavy metal uptake or stabilization [351]. Furthermore, compost or biochar as organic amendments ensure nutrient cycling by supplying macro and micro-nutrients, while providing carbon and energy to PGPR for their survival and sustained metabolic activity [352]. Similarly, bioaugmentation combined with biochar addition improved heavy metal (Cr, Zn, Fe, Al) remediation by increasing soil organic carbon and microbial activity, which enhanced metal immobilization and reduced bioavailability.

Integration of organic amendments with PGPRs is a highly effective strategy for mitigating heavy metal stress across a wide range of plant species, and soil types (Table 8). Combined application of organic amendments with microbes to various plant species consistently improved plant growth, enhanced antioxidant defenses, and regulated metal mobility, supporting both phytostabilization and phytoextraction depending on the remediation conditions. Improved plant performance under metal stress could be generally attributed to improved soil structure, nutrient availability, and pH buffering capacity of organic amendments such as compost, biochar, press mud, green manure, biogas slurry, and fermented sugar beet residues, which in turn may have bound with the metal and reduced their bioavailability. These benefits were further enhanced by introducing microbial inoculants, such as *Pseudomonas*, *Bacillus*, *Serratia*, *Rhizobium*, and arbuscular mycorrhizal fungi. In many cases, microbial activity increased nutrient uptake (particularly N and P), stimulated phytohormone production (e.g., IAA), and improved root architecture, leading to greater plant establishment and stress tolerance in contaminated soils [353,354,355,356]. Metal immobilization in the soil along with reduced root-to-shoot translocation of Pb, Cd, Cr, and Al resulted in metal detoxification in plants. The immobilization may be due to microbial biosorption, chelation, precipitation, and binding to organic matter. This phytostabilization resulted in reduced oxidative stress with decreased lipid peroxidation (MDA), and improved membrane stability [357]. On the other hand, arbuscular mycorrhizal fungi association with *Brassica juncea* and *Solanum nigrum* resulted in higher metal accumulation in shoots, highlighting their suitability for phytoextraction instead of phytostabilization [358,359]. In general, remediation varied depending on plant, metal, and amendment combinations, but microbes combined with organic amendments offered consistently synergistic and sustainable benefits, either immobilizing or extracting metals while improving soil health and plant production.

Organic amendments also improved the microbial functioning and diversity in the soil with improved soil enzymatic activity. Increases in dehydrogenase, phosphatase, urease, β-glucosidase, and protease activities were widely observed, indicating positive effects of treatments on soil biological functioning and nutrient cycling [355,372,376,377]. In multi-metal contaminated soils, organic amendments and microbial inoculants reshaped microbial community composition, often increasing the abundance of Proteobacteria, Actinobacteria, and Firmicutes, which are known for metal tolerance and promoting plant growth [359]. Importantly, remediation outcomes were context-dependent, varying with plant species, metal type, contamination level, and amendment characteristics. While microbial inoculation alone sometimes showed limited effects, its combination with organic amendments consistently produced synergistic responses.

Overall, the compiled studies in Table 8 exhibited that organic amendments with PGPR improves plant growth indicators up to four times [372] with decreased plant metal accumulation up to 78% [366]. Furthermore, combination of amendments improved plant physiological attributes such as antioxidant enzyme activities increased up to 163% [20], and soil microbial activity and enzyme functions improved >200% [360]. Overall, the evidence underscores that integrated amendment–microbe strategies provide a robust, ecologically sustainable approach for restoring metal-contaminated soils, offering flexibility to target either metal immobilization or extraction while simultaneously improving soil health and plant productivity.

## 7. Prospects and Challenges

### 7.1. Existing Challenges

There is huge gap in the application and success of bioremediation strategies in field compared to laboratory scale results largely due to the complex and variable biogeochemical processes operating under natural soil conditions. For instance, P fertilization enhances formation of insoluble pyromorphite with Pb^2+^ or Cd^2+^, limiting both the nutrient and the metal availability for plant uptake. In the rhizosphere, cations Ca^2+^, Mg^2+^, Fe^2+^, Zn^2+^, Mn^2+^, Cu^2+^, and NH_4_^+^ compete with heavy metals for uptake via root membrane transporters (e.g., *ZIP* and *NRAMP* families). On the other hand, nutrient overfertilization alters the soil’s microbial metabolic balance, leading secondary pollution risks. Heavy-metal-contaminated soil hosts stress-adapted oligotrophic bacteria, while high-nutrient inputs favor copiotrophic bacteria, which rapidly consume oxygen and create hypoxic zones. This can trigger the re-solubilization of reduced metal species (e.g., As(V) to As(III)) [382,383]. Excess nutrients, particularly P, act as dispersants for soil colloids and may also be transported into groundwater via colloid-facilitated transport [382].

Post-remediation handling of biomass requires careful thermal processing as pyrolysis can produce volatile metals like Hg- and As-posing air pollution risks [384]. In treating such wastes, hydrothermal carbonization in aqueous systems at relatively lower temperatures (180–250 °C) retains >90% of heavy metals in the solid “hydrochar” as oxides or sulfides [385]. For low-value metals like Pb and Cd, the cost of their extraction from ash is more expensive than their market value so the process is energy negative. On the other hand, high-value metals like Ni are likely to pay off the extraction cost [386]. Techno-economic analysis revealed that nutrients regulation is the only means of making phytoremediation competitive. It can reduce costs from $400,000–$1.2 M to $75,000–$100,000 per hectare and cut down remediation time from 50 to ~15 years, to meet the commercial viability threshold [10,387]. Life cycle assessments indicated that phytoremediation has a lower carbon footprint than excavation; however, ecotoxicity remains high unless biomass is properly treated via hydrothermal carbonization or controlled incineration [388].

### 7.2. Future Prospects

Bioremediation is a cost effective and energy efficient strategy; however, its effectiveness is constrained by the “biomass-concentration trade-off,” like amendments that mobilize metals can induce heavy metal toxicity while those that promote growth often immobilize them. Emerging evidence shows that nutritional regulation is evolving from a simple agronomic practice to a key intervention controlling bioremediation efficiency. We identify the following various frontiers that can drive the paradigm shift in bioremediation of metal contaminated soils.

#### 7.2.1. The Physiological Nexus: Nutritional Regulation and Heavy Metal Stress

Toxic metals enter plant cells through the same uptake systems that plants use for essential mineral nutrients, creating a nutritional-metal antagonism that can be exploited for remediation [389]. For instance, Cd^2+^ mimics Zn^2+^ and Fe^2+^, and its excessive uptake leads to oxidative stress, damaging the photosynthetic activity and reducing biomass production for Cd phytoaccumulation [390]. Thus, the challenge is to maintain sufficient Fe for plant functions without saturating the transporters with Cd^2+^. Similarly, As(V), compete with PO_4_^3−^ for high-affinity P transporters (PHT family). Excessive P supply suppresses As(V) uptake while P deficiency induces the expression of high-affinity transporters, dramatically increasing As accumulation [391]. Furthermore, intracellular heavy metal detoxification processes are linked with the assimilation of S and N. Upon exposure to metals like Cd^2+^ or Pb^2+^, plants redirect sulfur metabolism towards the glutathione [240]. Nitrogen fertilizer choice, NH_4_^+^ versus NO_3_^−^ provide a tool for altering the rhizosphere environment. This choice does not only change cation-anion balance but also significantly change rhizosphere pH with the acidification and alkalization affecting Cd, Zn, and Pb phytoextraction or phytostabilization [392].

#### 7.2.2. Controlled Release Nutrients and Biofertilizers

The theoretical understanding of nutrient–metal interactions does not explain the practical (temporal) nature of the interactions under conventional fertilizer use. However, controlled release, nano, and bifunctional fertilizers align nutrient supply with plant needs and regulate the metal concentration in soil solution [393]. For instance, chitosan-modified urea-formaldehyde (CSUF), a slow release N fertilizer, ensures consistent N supply to plant, while the NH_2_ and COOH groups on chitosan bind with Cu^2+^ and Cd^2+^ in the soil solution. The adsorption capacity of the CSUF gel beads for Cd^2+^, Cu^2+^, and Cr(VI) was 49.8, 6.63, and 40 mg/g, respectively. CSUF not only promoted mung bean dry (33.33%) and fresh biomass (47.83%) but also concomitantly reduced the Cd^2+^, Cu^2+^, and Cr(VI) content in the plant tissue by 89.28%, 92.08%, and 94.97%, respectively [394]. Nano-fertilizers with high surface area and properties improve heavy metal remediation. Fe-based nanomaterials increased plant biomass by 25% along with antioxidant enzyme activity, improving plant tolerance against heavy metal toxicity [395]. In addition, nano-hydroxyapatite (n-HAP) slowly released P to form stable pyromorphite with Pb^2+^ to reduce its bioavailability by 80%, thereby promoting root growth [396]. Similarly, Si nanoparticles reduced the apoplast metal bypass flow with the polymerization and also modulated the expression of antioxidant enzymes to withstand higher internal metal loads [397]. Combined application of controlled release, nano, and bifunctional fertilizers with PGPR further improve their remediation efficiency. Inoculation of *Bacillus* strains to *Solanum nigrum* increased shoot biomass by 136–170% and Cd uptake by 228–281% [398]. Foliar application of *Pseudomonas fluorescens* on *Sedum alfredii* increased its biomass by 39% and Cd removal by 107% [399]. Similarly, endophytic *Pseudomonas* E3 increased Cd extraction efficiency by 40.26% in *Solanum nigrum* and its biomass by 20.47% [400]. Integration of these approaches provides a promising and sustainable future strategy for improving heavy metal phytoremediation through optimizing nutrient delivery, metal mobilization/stabilization, and plant–microbe interactions.

#### 7.2.3. Synthetic Biology and Genetic Engineering

Regulation of the plant internal metabolic processes to mitigate heavy metal toxicity require changes in the expression of the metal acquisition and detoxification processes. Targeting key metal transporter families, such as ZIP, NRAMP, CDF, YSL, MFS, HMA, VIT, and CAX, can enhance plant phytoremediation efficiency. Transgenic tobacco with the upregulation of SmZIP transporter accumulated higher Cd content in leaves and stems with higher growth rate compared to wild type [401]. Similarly, over expression of ATP-binding cassette (ABC) transporter gene in *Brassica juncea*, *Arabidopsis* and *Populus* spp. increased plant growth as well as Cd, Pb and Hg accumulations [402,403]. Furthermore, the expression of bacterial *polyphosphate kinase* (*ppk*) genes in plants results in an inorganic poly-phosphate chain that sequester metals [404]. Engineered *Pseudomonas* strains were able to produce high affinity Cd^2+^-binding siderophores with increased Cd uptake [405].

A major drawback of using genetically modified organisms is the potential risk of gene flow into natural ecosystem. To address this concern, efforts have been directed to design robust biocontainment strategies, such as engineering microorganisms that depend on synthetic amino acids for survival, preventing them from persisting in natural conditions [406,407,408]. In addition, kill switches with engineered genetic circuits triggers cell lysis under specific environmental conditions which ensures that engineered remediators persist only as long as the pollution remains. CRISPR-based kill switches have demonstrated high genetic stability and efficiency in microbes such as *Escherichia coli*, with reduced mutant escape and improved biocontainment [409]. Furthermore, cisgenesis provides an opportunity to transfer genes from one genotype to another of the same species such as insertion of a transporter gene from a wild genotype to a fast-growing genotype, thereby avoiding the transgenic label and potentially accelerating adoption of such plant for phytoremediation purposes. In a complementary approach, chromosome segment substitution lines have been successfully employed to design rice cultivars with low grain cadmium accumulation while preserving agronomic traits, demonstrating a precision breeding route for metal-safe crops [410].

#### 7.2.4. AI, Multi-Omics, and Precision Remediation

Artificial Intelligence (AI) is increasingly becoming a powerful tool required to simplify the complex interactions among plant genetics, soil chemistry, and climate [411]. AI algorithms are now being used to interpret the massive datasets related to ionomic, proteomic, metabolomic and transcriptomic. As a result, complex genetic networks and regulatory pathways that influence plant characteristics and environmental reactions can be easily identified [412]. Machine learning models such as Random Forest (RF), Extreme Gradient Boosting (XGBoost), and Artificial Neural Networks (ANNs) can be used to predict heavy metal removals by combining soil pH, moisture content, plant biomass and initial metal concentration to give highly accurate remediation predictions [413]. Integration of ionomic data with genomic sequencing across diverse plant species enables AI-driven genome-wide association (GWAS) studies, allowing a model to identify complex “Gene Network” clusters associated with metal tolerance [408]. Ultimately, these technologies will lead us to the creation of “Digital Twins” which allow for the simulation of thousands of in silico scenarios to ascertain likely remediation outcomes and assist with decision-making [414,415,416].

## 8. Conclusions

Microbial processes including biosorption, bioaccumulation, enzymatic reduction, precipitation, and biotransformation of toxic metals, are affected by organic carbon source availability and types. Biosorption by the cell wall functional groups and exopolysaccharides effectively immobilizes metals, often exceeding 100 mg/g in some *Bacillus*, *Enterobacter*, *Klebsiella*, *Lysinibacillus*, *Pseudomonas*, and *Rossellomorea* strains. While quinone-rich amendments, humic substances and dissolved organic matter favor metal reduction through microbial metabolism and electron shuttling processes, microbial driven detoxification processes are governed by the balance in the C:N:P ratio. Low N availability restricts microbial growth and enzyme synthesis needed for metal detoxification. Phosphorus fertilization affects phosphate-solubilizing microorganisms and the formation of insoluble metal–phosphate complexes with Pb, Cd, and Zn. Sulfur transformation, especially sulfate reduction by microbes, results in insoluble metal sulfides and effectively precipitates (>90%) Cd^2+^, Pb^2+^, Ni^2+^, Fe^2+^, Mn^2+^, Cu^2+^, and Cr(III). Generally, consortia achieve higher metal precipitations (e.g., Ni^2+^ > 400 mg/L and Fe^2+^ > 800 mg/L) compared to individual microbial species. The summary for decision-support framework linking heavy metals, remediation strategies, chelator-assisted phytoextraction, and nutrient management is presented in Table S2.

Phytoextraction efficiency is associated with NH_4_^+^ -N, which enhances As, Cd acquisition by decreasing soil pH. In addition, phytoextraction efficiency of *Brassica juncea*, *Zea mays*, and *Panicum virgatum* can be enhanced 30–40-fold by using metal chelators such as EDTA. However, high mobility and limited biodegradability of EDTA–metal complexes pose a significant environmental risk; henceforth biodegradable chelates with moderate effects on plant metal uptakes can be used as environment friendly alternative. Contrarily, amendments such as NO^−^_3_-N, PO_4_^3−^, and SO_4_^2−^ containing fertilizers enhance metal phyostabilization. These benefits can further be enhanced by introducing microbial inoculants, such as *Pseudomonas*, *Bacillus*, *Serratia*, *Rhizobium*, and arbuscular mycorrhizal fungi. Plant-growth-promoting rhizobacteria facilitate bioremediation processes through N_2_ fixation, P solubilization, siderophore release and phytohormone production leading to improved root architecture, plant biomass and metal tolerance. Evidence strongly supports the concept of integrated remediation through combining microbial processes, plant-based extraction, with appropriate soil amendments offering the most effective pathway for sustainable heavy metal remediation. Advancing such integrated approaches with the inclusion of biotechnological interventions will further strengthen the heavy metal phytoremediation and contribute to restoring the ecological integrity of contaminated ecosystems in a rapidly industrializing world.

## Figures and Tables

**Table 1:** The effectiveness of heavy metal removal by microbes and detoxification mechanisms. Metal sorption capacities demonstrated by various microorganisms.

Metal	Microbial Species (Strain)	Mechanism	Removal (%) or *Q*_max_	Citation
Cd	*Bacillus* sp. MC3B-22	Biosorption (EPS)	*Q_max_* ≈ 141 mg/g	[77]
Cd	*Microbacterium* sp. MC3B-10	Biosorption (EPS)	*Q_max_* ≈ 97 mg/g	
Cd	*Bacillus cereus* RC-1 on magnetic biochar	Biosorption/adsorption + bioaccumulation	*Q_max_* ≈ 93.02 mg/g	[78]
Cd	*Serratia bozhouensis* CdIW2	Biosorption	*Q_max_* ≈ 65.79 mg/g	[79]
Cd	*Agrobacterium tumefaciens* S12	Biosorption	*Q_max_* ≈ 58 mg/g	[80]
Pb	*Lactobacillus brevis*	Biosorption	*Q_max_* ≈ 53.63 mg/g	[81]
Pb	*Oceanobacillus profundus* KBZ 3–2	EPS ion exchange and complexation	97% removal	[82]
Pb	Acid-tolerant lactic strains CN-011 and CN-005	Biosorption	≈85.95–86.78% removal	[83]
Pb	*Bacillus subtilis*	Biosorption	*Q_max_* ≈ 61.8 mg/g (≈96.1%)	[84]
Pb	*Enterobacter chuandaensis* DGI-2	Biosorption/bioaccumulation	*Q_max_* ≈ 102.95/98.61 mg/g	[85]
Pb	*Methylobacterium hispanicum* EM2	Surface binding	*Q_max_* ≈ 79.84 mg/g	[86]
Pb	*Klebsiella* sp. 3S1	Biosorption	*Q_max_* ≈ 140.19 mg/g	[87]
Cr	*Mycobacterium* sp.	Biosorption	*Q_max_* ≈ 42.81/26.56 mg/g	[88]
Cr	*Ochrobactrum intermedium* LBr	Biosorption	*Q_max_* ≈ 51.96/10.94 mg/g	[89]
	*Cupriavidus metallidurans* CH34		*Q_max_* ≈ 32.63/47.79 mg/g	
Cr	*Cronobacter muytjensii* KSCAS2	Biosorption	*Q_max_* ≈ 72.45 mg/g	[90]
Cr	*Pseudomonas stutzeri*	Biosorption	*Q_max_* ≈ 27.47 mg/g	[91]
Cr	*Pseudomonas alcaliphila* NEWG-2	Biosorption	*Q_max_* ≈ 10 mg/g	[92]
Cr	*Pseudomonas stutzeri* YC-34	EPS adsorption	*Q_max_* ≈ 26.32 mg/g	[93]
Cr	*Brachybacterium paraconglomeratum* ER41	Biosorption + reduction	*Q_max_* ≈ 33.814 mg/g	[94]
Cr	*Pseudomonas koreensis* (immobilized)	Biofilm adsorption	*Q_max_* ≈ 166 mg/g	[95]
Ni	*Lysinibacillus* sp. BA2	Cell surface biosorption	*Q_max_* ≈ 238.04/196.32 mg/g	[96]
Ni	*Pseudomonas* sp.	Biosorption	*Q_max_* ≈ 27.2 mg/g	[97]
	*Bacillus cereus*		*Q_max_* ≈ 27.4 mg/g	
	*Pseudomonas fungorum*		*Q_max_* ≈ 27.3 mg/g	
	*Bacillus fungorum*		*Q_max_* ≈ 27.37 mg/g	
	*Bacillus safensis*		*Q_max_* ≈ 27.40 mg/g	
	*Bacillus subtilis*		*Q_max_* ≈ 27.33 mg/g	
Hg	*Pseudomonas* sp. B50A	MerA reduction	86% removal	[98]
Hg	*Bacillus licheniformis*	EPS biosorption	*Q_max_* ~200 µg/g biomass	[99]
Hg	*Pseudomonas aeruginosa* FZ-2	merA expression	≈91% removal	[100]
Hg	Mixed Yellow River isolates	Mer operon reduction	≤0.05 mg/L–10 mg/L	[101]
Hg	*Bacillus* sp. LBA119	Volatilization + biosorption	97.3% removal	[102]
Hg	*Rheinheimera metallidurans*	Mercuric reductase	≈92% removal	[103]
Hg	*Pseudomonas putida* MERCC_1942	Biosorption + biotransformation	36.8 pmol Hg/cell/h	[104]
Hg	*Stenotrophomonas* sp. INV PRT0231	Mercury removal efficiency	8.4 mg Hg/g biomass/h	[105]
As	*Acinetobacter junii*	As oxidation	639 µg extracellular and 618 µg intracellular	[106]
	*Bacillus cereus*		667 µg extracellular and 414 µg intracellular	
As	*Delftia tsuruhatensis* K7	Oxidation + adsorption	92.4% oxidation	[107]
As	*Pseudomonas* sp. SMS11	Biosorption + oxidation	555 mg/g surface binding	[36]
As	*Acinetobacter* sp. Sp2b	Biosorption	*Q_max_* ≈ 20.1 mg/g	[108]
Cu	*Bacillus pumilus* OQ931870	Biosorption	*Q_max_* ≈ 11.876 mg/g	[109]
	*Bacillus subtilis* OQ931871		*Q_max_* ≈ 19.88 mg/g	
Cu	*Rossellomorea* sp. ZC255	Chemisorption	*Q_max_* ≈ 253.4 mg/g	[110]
Cu/ Mn	*Providencia* sp. LLDRA6	Biosorption + bioleaching	Cu 91.25%, Mn 56.13%	[111]
Mn	*Serratia marcescens* QZB-1	Biosorption + oxidation	91.8% Mn removal	[60]
Mn	*Bacillus wiedmannii*	Biosorption	91.96% Mn removal	[112]

Note: “≈” Represents estimated value from published figures using visual digitization.

**Table 2:** Efficiency of sulfate-reducing bacteria/consortia in removing the heavy metals/metalloids under varying conditions of metal type, concentrations, and pH.

Microorganism/Consortium	Target Metal Concentration (mg/L)	Removal Efficiency (%)	Optimal pH	Citation
*Desulfovibrio desulfiricans*	Fe: 463	Fe ≥ 99.9	7.8–8.3	[144]
	Cu: 76	Cu ≥ 99.9		
	Cd: 58	Cd ≥ 99.9		
	Zn: 118	Zn ≥ 99.9		
	Mn: 79	Mn = 42.1–99.3		
Mixed SRB biomass (including *Desulfovibrio*)	Cd: 5–10	Cd: 94.8	~7.5–8	[139]
	Ni: 5–10	Ni: 97		
	Pb: 5–10	Pb: 94.4		
	Zn: 5–10	Zn: 94.6		
	Cu: 25–50	Cu: 98.9		
	Fe: 10–25	Fe: 93.9		
Indigenous SRB consortium (dominated by *Desulfosporosinus* sp.)	As: ~79.5	As = 100	4.5–5.0	[145]
	Zn: ~15.0	Zn = 100		
Indigenous bacterial culture + *Desulfovibrio desulfuricans*	Zn: 174	100	5.8	[146]
SRB-enriched sludge (dominant genus *Desulfovibrio*, 43.3%)	Ni: 441	≈99–100	7	[140]
SRB consortium (*Desulfovibrio idahonensis*, *Desulfotomaculum* sp.)	Pb: 50	>95	6.5–7.0	[147]
	Zn: 100	>90		
*Desulfovibrio vulgaris* + emulsified corn oil	Cd: 9.7	Cd ≈ 99	~7	[148]
	Mn: 20	Mn ≈ > 90		
	Zn: 186.9	Zn ≥ 96.8		
	Fe: 48.9	Fe = 100		
*Desulfovibrio desulfuricans*	Cd: 30	>95	5–7	[149]
*Desulfovibrio* sp. ZHS8	Cd: 20	99	5.0–7.0	[150]
*Desulfobulbus propionicus*	Cd: 10–100	Cd = 45.7–98.7	8	[24]
	Pb: 30–150	Pb = 24.9–99.9		
*Desulfovibrio desulfuricans* CSU_dl	Sb(III): 30	98.09	6	[151]
	Sb(V): 45	94.89	7	
*Desulfovibrio* sp. W21	Fe: 200–800	97	4.5–9.0	[152]

Note: “≈” Represents estimated value from published figures using visual digitization.

**Table 3:** Role of organic amendments in microbial- and consortia-based microbial Cr(VI) reduction and detoxification efficiency affected by organic carbon source type, its concentration, microorganisms and Cr(VI) concentration.

Microorganism/Consortium	Organic Amendment	Amendment Concentration	Initial Cr(VI) Conc.	Reduction Efficiency (%)	Mechanistic Information	Citation
*Halomonas smyrnensis* KS802	Galactose	4%	2 mM	100%	Microbial reduction of Cr(VI) to Cr(III) and its binding/precipitation to cell surface	[16]
*Arthrobacter* sp.	Lactate	15 mM	50 µM	80%	Metabolic (enzyme-mediated), linked to cell growth, with the partial abiotic reduction	[160]
*Geobacter sulfurreducens*	Anions (SO_4_^2−^) and dissolved organic matter (AQDS)	2.5, 5 and 20 mM	96 µM	87% 94% 100%	Electron transfer increased from 1.48 to 3.00 meq/L/h with AQDS concentrations increasing from 0 to 20 mM which accelerated microbial reduction.	[161]
*Stenotrophomonas* sp. WY601	Lactose, fructose, glucose	2%	300 mg/L 500 mg/L 1000 mg/L 1500 mg/L 2000 mg/L 2500 mg/L	≈90% ≈67% ≈42% ≈33% ≈31% ≈28%	Cr(VI) reductase found within the extracellular membrane or adsorbed to its surface mainly contributed to reduction processes.	[153]
*Bacillus* sp. M6	Glycerol, glucose	0.1 mM 0.1 mM	20 mg/L	>69% >65%	Both extracellular polymeric substance/membrane-mediated mechanisms and internal enzymes contributed to Cr(VI) reduction.	[162]
*Flexivirga alba* ST13T	Sugarcane molasses	0.4%	0.5–0.6 mg/L	>95%	In addition to bacterial Cr(VI) reduction, molasses contributes by forming the Cr(VI)–polyphenol complex especially at acidic pH.	[163]
*Bacillus* sp. CRB-B1	Fructose, glucose	10 g/L 10 g/L	100 mg/L	>89% >76%	Cr(VI) reduction mainly occurred extra-cellularly and mediated by extracellular reductase.	[164]
*Geobacter sulfurreducens*	Dissolved organic matter (DOM) from composted sewage sludge	135 mg C/L of sludge DOM	13.52 mg/L	55.4%	In addition to microbial reduction, humic substances from DOM acted as electron shuttle and promoted its immobilization on microbial cells.	[165]
*Azospirillum*, *Pseudarcicella*	Lactate	336.2 mg/L	1000 µg/L	~81.7%	14 days; fermentation-driven electron donors	[166]
*Pseudomonas aeruginosa* (PA)	Fe-rich sludge biochar (prepared at 300 °C)	1 g/L	10 mg/L	80%	Biochar P precipitates Cr; cellular proteins assist reduction	[167]
*Bacillus cereus* RC	Glucose + ferric citrate	Glucose 3 g/L; ferric citrate 1.5 g/L	50 mg/L	~83%	Reduction kinetic enhancement rather than final removal	[168]
*Geobacter sulfurreducens*	Fumarate, pyruvate, and oxalate,	0.05 M 0.05 M 0.05 M	600 μM	98% 97% and 96%	Electron-shuttle-mediated reduction	[169]
Native soil microbiota (Not specified)	Poultry manure biochar modified with chitosan	5% (*w*/*w*)	100 mg/kg	>79%	PAHs in biochar donate protons for Cr(VI) reduction	[170]
	Sheep manure biochar modified with chitosan	5% (*w*/*w*)	100 mg/kg	>89%		
Microbial consortia (Not specified)	H_2_:CH_4_ (1:1 ratio)	Bioreactor was supplied with H_2_:CH_4_ (1:1 ratio) through a needle connecting with gas cylinders for 30 min	10 mg/L	>95%	Both H_2_ and CH_4_ as electron donors synergistically contributed to microbial Cr(VI) reduction, hydrogenotrophic bacteria and CH_4_-metabolizing microorganisms.	[171]
Microcosms (Autochthonous soil microbes–*Shewanella oneidensis* detected but not dominant)	Yeast extract	200 mg/L	1000 µg/L	100%	Within 7 days; organic carbon stimulated reduction	[172]
	Lactate	3 mM	1000 µg/L	≈90%	After 11 days; sodium lactate electron donor	
Mixed indigenous groundwater microbes	Molasses	Not specified	25 mg/L	100%	Molasses improved electron transport capacity	[173]
Indigenous microbial consortium	Yeast extract	200 mg/L	134 µg/L	100%	Within 7 days 100% reduction was achieved	[174]
	Polyhydroxybutyrate	180 mg/L	70 µg/L	100%	Needed 21 days to achieve 100% reduction	
Not specified	FeS_2_/biochar composite + oxalic acid	5 mM	50 mg/L	>96%	Synergistic abiotic–microbial reduction	[175]
Native bacterial consortia (Dominated by *Shewanella*, *Vogesella*, and *Acinetobacter*	Yeast extract	200 mg/L	1000 µg/L	100%	14 days; extracellular electron shuttle dominant	[166]

Note: “≈” Represents estimated value from published figures using visual digitization.

**Table 4:** Heavy metal hyperaccumulator plant species belonging to various families with their capacity to accumulate metal(s) in the shoot (concentration ranges µg/g DW).

Plant Family	Plant Species	Metal	Concentration (µg/g DW)	Citation
Brassicaceae	*Alyssum murale*, *Alyssum corsicum*, *Alyssum bertolonii*,	Ni	30,000	[181,189]
Phyllanthaceae	*Phyllanthus rufuschaneyi*, *Phyllanthus* cf. *securinegoides*, *Phyllanthus balgooyi*,		2413–25,000	[190,191]
	*Actephila alanbakeri Welzen and Ent*		8178	[192]
	*Emblica rufuschaneyi (Welzen*, *R.W.Bouman &* * Ent) R.W.Bouman*		24,000	
	*Glochidion brunneum*, *Glochidion* sp. *‘bambangan’ Glochidion* sp. *‘nalumad’*, *Glochidion* cf. *lanceisepalum*, *Glochidion rubrum*, *Glochidion sericeum*, *Glochidion mindorense*		1400–7200	[191]
Violaceae	*Rinorea linearifolia Latiff*		7850	[192]
	*Rinorea javanica*		6926	[191]
	*Rinorea bengalensis*		7510	
Meliaceae	*Walsura pinnata Hassk*		1897	[192]
Salicaceae	*Xylosma luzonensis Clos*		5510	
Brassicaceae	*Noccaea caerulescens*, *Noccaea ochroleuca*, *Noccaea praecox*	Zn	14,000–29,000	[193,194]
	*Arabidopsis halleri*		32,000	[195]
Crassulaceae	*Sedum alfredii* (Zn ecotype)		10,000	[196]
Dichapetalaceae	*Dichapetalum gelonioides*		10,730	[197]
Brassicaceae	*Noccaea caerulescens*	Cd	3500	[198]
	*Arabidopsis halleri*		>1000	[199,200]
Crassulaceae	*Sedum alfredii*, *Sedum plumbizincicola*		Up to 9000	[196,201]
Pteridaceae	*Pteris vittata*, *Pteris cretica*, *Pteris multifida*, *P. longifolia and P. umbrosa*	As	1500–23,000	[202,203]
	*Pityrogramma calomelanos*		>8000	[204]
	*Ocimum tenuiflorum* L.	Cu	Up to 2265	
	*Haumaniastrum robertii*		Up to 6159	[205]
Commelinaceae	*Commelina communis*		1000–3000	[206]
Crassulaceae	*Crassula helmsii*		>9000	[207]
Euphorbiaceae	*Acalypha cupricola* Robyns		Up to 2890	[208]
Fabaceae	*Tephrosia villosa* Pers.		Up to 1858	[206]
Asteracea	*Anisopappus chinensis*		Up to 2800	[205,209]
	*Vernoniastrum latifolium* (Steetz) H. Rob.		Up to 1942	[208]
Caryophyllaceae	*Silene cobalticola P.A. Duvign. and Plancke*		Up to 1660	[205]
Poaceae	*Polypogon fugax Nees ex steud.*		Up to 4012	[210]
Verbenaceae	*Clerodendrum infortunatum* L.		2280	[206]
Asteracea	*Anisopappus chinensis*	Co	Up to 1300	[209]
Lamiaceae	*Aeollanthus subacaulis*		Up to 1900	[211]
Malvaceae	*Triumfetta welwitschii*		Up to 1971	[208]
Phyllanthaceae	*Glochidion* cf. *sericeum (Blume)*		Up to 1310	[191]
Commelinaceae	*Cyanotis longifolia Benth. var. longifolia*		Up to 1697	[205]

**Table 5:** Chelate-assisted heavy metal uptake in plants and its role in phytoextraction of heavy metals by plants.

Plant Species	Heavy Metal	Soil/Growth Conditions	Chelator (Dose)	Shoot HM Concentration (mg/kg DW)	Enhancement Factor	Citation
*Lantana camara*	Cd	Garden soil (considered as control) compared with 20%-added fly ash soil. Fly ash contains heavy metal; Cd (1.01–2.05), Cr (5.05–11.35), Cu (16.6–31.15), Mn (94.95–141.55), Ni (1.85–5.55), and Pb (9.8–24.5).	EDDS 10 mmol/kg	≈35 (5 in control)	7.0×	[252]
	Cr			≈75 (10 in control)	7.5×	
	Cu			≈65 (8 in control)	8.1×	
	Mn			≈65 (15 in control)	4.3×	
	Ni			≈30 (3 in control)	10.0×	
	Pb			≈28 (3 in control)	9.3×	
*Helianthus annuus* L.	Cd	Plants were grown in pots containing soil with Cd added at 15 mg/kg. No EDDS amendment considered as control	EDDS (2.5 mmol/kg)	≈37 (18 in control)	2.1×	[253]
			EDDS (5.0 mmol/kg)	≈70 (18 in control)	3.9×	
			EDDS (7.5 mmol/kg)	≈55 (18 in control)	3.1×	
	U	Plants were grown in pots containing soil with U added at 15 mg/kg. No EDDS amendment considered as control	EDDS (2.5 mmol/kg)	≈0.50 (0.21 in control)	2.4×	
			EDDS (5.0 mmol/kg)	≈0.88 (0.21 in control)	4.2×	
			EDDS (7.5 mmol/kg)	≈1.00 (0.21 in control)	4.8×	
*Brassica juncea*	Cr	Study was conducted at Suva, located on the southeast coast of the island of Viti Levu, in the Republic of Fiji. Multi-meal spiked soil containing Cr (159 mg/kg), Zn (128.50 mg/kg, Cd (44.70 mg/kg), Pb (312.60 mg/kg), Ni (143.90 mg/kg), and Cu (264.80 mg/kg)	EDDS 5 mmol/kg	≈70 (25 in no Chelate)	2.8×	[254]
	Zn			≈120 (60 in no Chelate)	2.0×	
	Cd			≈60 (20 in no Chelate)	3.0×	
	Pb			≈310 (200 in no Chelate)	1.6×	
	Ni			≈170 (90 in no Chelate)	1.9×	
	Cu			≈40 (27 in no Chelate)	1.5×	
*Brassica rapa*	Cr			≈45 (25 in no Chelate)	1.8×	
	Zn			≈110 (90 in no Chelate)	1.2×	
	Cd			≈30 (20 in no Chelate)	1.5×	
	Pb			≈180 (130 in no Chelate)	1.4×	
	Ni			≈100 (70 in no Chelate)	1.4×	
	Cu			≈30 (28 in no Chelate)	1.1×	
*Solanum* * nigrum*	Cd	Soil spiked with different Cd treatments (12 and 48 mg/kg soil)	EDDS 10 mM	8.38 (4.08 in +Cd12)	2.1×	[15]
				29.16 (16.11 in +Cd48)	1.8×	
*Brassica napus* L.	Pb	Hoagland’s nutrient solution; Pb 50 µmol/L and Pb 100 µmol/L	EDTA 2.5 mM	≈1850 (1200 in +Pb50)	1.5	[255]
				≈3200 (2400 in +Pb100)	1.3	
	Cu	Hoagland’s nutrient solution; Cu 50 µmol/L and Cu 100 µmol/L	EDTA 2.5 mM	≈300 (200 in +Cu50)	1.5×	[256]
				≈600 (420 in +Cu100)	1.4×	
*Solanum nigrum* L.	Cd	Cd-contaminated soil (0, 5, 10, 25, 50, 100, 200 mg/kg soil)	EDTA (1.5 and 5 mmol/kg soil)	150–160 (135 control)	≈1.11–1.19×	[257]
*Iris halophila Pall.*	Pb	Dexing Pb Mine, Jiangxi Province, China, Pb mine tailings with available Pb 706.93 mg/kg	EDTA 0.5 mmol/kg	≈1025 (715 in control)	1.4×	[258]
			EDTA 2 mmol/kg	≈1543 (715 in control)	2.2×	
*Petunia hybrida* L.	Cd	Hydroponic system with (100 μM) Cd, Cr, Cu, and Ni. Metals were tested individually	EDTA (2.5 mM)	~1350 (850 in +Cd)	~1.6×	[259]
	Cr			~1600 (600 in +Cr)	~2.7×	
	Cu			~1300 (450 in +Cu)	~2.9×	
	Ni			~800 (300 in +Ni)	~2.7×	
*Nicotiana alata* L.	Cd	Hydroponic study with 100 mM metal contamination	EDTA (2.5 mM)	≈875 (420 in +Cd)	2.1×	[260]
	Cr			≈1450 (575 in +Cr)	2.5×	
	Cu			≈250 (145 in +Cu)	1.7×	
	Ni			≈1250 (775 in +Ni)	1.6×	
	Pb			≈1400 (1200 in +Pb)	1.17×	
*Corchorus capsularis* L.	Cu Cu	Hydroponic nutrient solution; Cu 50 µmol/L and Cu 100 µmol/L	EDTA of 3 mM	33 (26 in +Cu50)	1.3×	[261]
				45 (40 in +Cu100)	1.1×	
*Zea mays* L.	Pb	Zn/Pb deposits in the Irankouh-Iran mining site, with a Total Pb and Zn, were 567.5 and 245.0 mg/kg, respectively. The available Pb and Zn concentrations were 35.0 and 34.67 mg/kg, respectively	EDTA (8 mmol/kg)	416 (10.6 in control)	39.3×	[262]
	Zn			530.4 (100.9 in control)	5.3×	
*Zea mays* L.	Cd	Soil contaminated with and its concentration before the start of the experiment was 6.53 mg/kg	EDTA (3 mmol/kg)	≈6.45 (2.55 in control)	2.5×	[250]
*Panicum virgatum* L.	Pb	Highly contaminated soil from the former Superfund site in Cedartown, GA. Pb concentrations (up to 260,000 mg/kg)	EDTA (1 mM)	3315 (82 in control)	≈3.8×	[263]
*Indocalamus decorus Q. H. Dai*	Pb	The soil was adequately mixed with CH_3_COOH)_2_Pb·3H_2_O to obtain contaminated soil with a Pb concentration of 1500 mg/kg	EDTA (1500 mg/kg soil)	≈430 (420 in control)	1.02×	[264]
*Sedum hybridum*	Cd	Garden soil, peat and vermiculite mixture (2:2:1) at the greenhouse of Beijing Forestry University with Cd and Pb being 45.4 mg/kg and 1342 mg/kg, respectively	EDTA 6 mmol/kg	52.5 (32.5 in control)	1.7×	[265]
	Pb			≈1050 (780 in control)	1.3×	
*Brassica juncea* L.	Pb	Multi-metal contaminated industrial wastewater was used to irrigate soil. The metal concentrations were 12.36 Pb, 10.64 Ni, 30.45 Zn and 6.5 Hg mg/L	EDTA (4 mM/kg soil)	≈900 (20–25 in control)	36–45×	[248]
	Zn			≈750 (20–22 in control)	34–38×	
	Ni			≈450 (10–12 in control)	37–45×	
	Hg		EDTA (3 mM/kg soil)	≈190 (5–7 in control)	27–38×	
*Marrubium cuneatum*	Pb	Soil polluted with Zn, Pb, and Cd from the Angouran Pb–Zn mine site, Total Zn, Pb, and Cd concentrations were 568.42, 472, and 6.85 mg/kg, respectively	EDTA (5 mmol/kg)	~143 (46 in control)	~3.11×	[246]
	Cd			11.49 (2.88 control)	~3.99×	
	Zn			418(210)	~1.99×	
*Stipa arabica*	Pb			~115 (17.4 in control)	~6.33×	
	Cd			10.75 (6.65 control)	~1.62×	
	Zn			365 (260 control)	~1.40×	
*Verbascum speciosum*	Pb			~325 (68 in control)	~4.76×	
	Cd			13.50 (6.47 control)	~2.09×	
	Zn			400 (240 control)	1.67×	
*Portulaca* * oleracea*	Cd	Multi-metal contaminated soil from the town of Lavrio-Greece renowned for its historical (now discontinued) mining activities. Pseudo-total metal contents; Cd = 101.9, Pb = 26,526.4, Zn = 17,652.6, and Cu = 181.6 mg/kg	EDTA at 20 mmol/kg	≈2.55 (1.21 in control)	2.1×	[266]
	Cu			≈5.18(3.52 in control)	1.5×	
	Pb			≈187.66 (53.73 in control)	3.5×	
	Zn			≈366.82 (180.17 in control)	2.0×	
	Cd		EDTA at 40 mmol/kg	≈3.84 (1.21 in control)	3.2×	
	Cu			≈5.19 (3.52 in control)	1.5×	
	Pb			≈280.35 (53.73 in control)	5.2×	
	Zn			≈464.04 (180.81 in control)	2.6×	
*Portulaca* * oleracea*	Cd	Cd contamination 40, 80 and 100 mg/kg soil	EDTA (0.1, 0.3 mg/kg soil)	187–222 (126 in control)	1.5–1.76×	[267]
*Indocalamus decorus Q. H. Dai*	Pb	Soil was mixed with CH_3_COOH)_2_Pb·3H_2_O to obtain a Pb concentration of 1500 mg/kg soil	EDTA + GLDA (750 + 750 mg/kg soil)	≈400 (420 in control)	0.95×	[264]
			EDTA + NTA (750 + 750 mg/kg soil)	≈640 (420 in control)	1.5×	
*Solanum* * nigrum*	Cd	Soil spiked with different Cd treatments (12 and 48 mg/kg soil)	EGTA 10 mM	8.83 (4.08 in +Cd12)	2.2×	[15]
				42.76 (16.11 in +Cd48)	2.7×	
*Zea mays* L.	Pb	Zn/Pb deposits in the Irankouh-Iran mining site with a Total Pb and Zn were 567.5 and 245.0 mg/kg, respectively. The available Pb and Zn concentrations were 35.0 and 34.67 mg/kg, respectively	GLDA (8 mmol/kg)	398 (10.6 in control)	37.5×	[262]
	Zn			718.9 (100.9 in control)	7.1×	
*Zea mays* L.	Cd	Soil contaminated with and its concentration before the start of the experiment was 6.53 mg/kg	GLDA (3 mmol/kg)	≈7.68 (2.55 in control)	3.0×	[250]
*Brassica juncea*	Cr	Study was conducted at Suva, located on the southeast coast of the island of Viti Levu, in the Republic of Fiji. Multi-meal spiked soil containing Cr (159 mg/kg), Zn (128.50 mg/kg, Cd (44.70 mg/kg), Pb (312.60 mg/kg), Ni (143.90 mg/kg) and Cu (264.80 mg/kg)	GLDA 3 mmol/kg	≈60 (25 in no Chelate)	2.4×	[254]
	Zn			≈100 (60 in no Chelate)	1.7×	
	Cd			≈35 (20 in no Chelate)	1.8×	
	Pb			≈315 (200 in no Chelate)	1.6×	
	Ni			≈180 (90 in no Chelate)	2.0×	
	Cu			≈35 (27 in no Chelate)	1.3×	
*Brassica rapa*	Cr			≈60 (25 in no Chelate)	2.4×	
	Zn			≈95 (90 in no Chelate)	1.1×	
	Cd			≈40 (20 in no Chelate)	2.0×	
	Pb			≈175 (130 in no Chelate)	1.3×	
	Ni			≈75 (70 in no Chelate)	1.1×	
	Cu			≈18 (28 in no Chelate)	0.6×	
*Indocalamus decorus Q. H. Dai*	Pb	Soil was mixed with CH_3_COOH)_2_Pb·3H_2_O to obtain a Pb concentration of 1500 mg/kg	GLDA (1500 mg/kg soil)	≈510 (420 in control)	1.2×	[264]
*Solanum nigrum*	Cd	Soil samples from the grassland of Jiangnan University in Wuxi, Jiangsu Province. Soil Cd contents were set at (20 and 40 mg/kg)	GLDA 3.0 mmol/kg	≈88 (65 in +Cd20)	1.4×	[268]
				≈125 (82 in +Cd40)	1.5×	
*Sedum hybridum*	Cd	Garden soil, peat and vermiculite mixture (2:2:1). at the greenhouse of Beijing Forestry University with Cd and Pb being 45.4 mg/kg and 1342 mg/kg, respectively	GLDA 6.0 mmol/kg	≈41.5 (32.5 in control)	1.3×	[265]
	Pb			≈1300 (780 in control)	1.6×	
*Zea mays* L.	Cd	Soil contaminated with and its concentration before the start of the experiment was 6.53 mg/kg	IDSA (3 mmol/kg)	≈5.46 (2.55 in control)	2.1×	[250]
*Lantana camara*	Cd	Garden soil (considered as control) compared with 20%-added fly ash soil. Fly ash contains the heavy metal concentrations Cd (1.01–2.05), Cr (5.05–11.35), Cu (16.6–31.15), Mn (94.95–141.55), Ni (1.85–5.55), and Pb (9.8–24.5)	MGDA 10 mmol/kg	≈50 (5 in control)	10.0×	[252]
	Cr			≈55 (5 in control)	11.0×	
	Cu			≈60 (5 in control)	12.0×	
	Mn			≈55 (15 in control)	3.7×	
	Ni			≈45 (5 in control)	9.0×	
	Pb			≈30 (5 in control)	6.0×	
*Zea mays* L.	Pb	Zn/Pb deposits in the Irankouh-Iran mining site, with a Total Pb and Zn, were 567.5 and 245.0 mg/kg, respectively. The available Pb and Zn concentrations were 35.0 and 34.67 mg/kg, respectively	MGDA (8 mmol/kg)	416 (10.6 in control)	39.3×	[262]
	Zn			798.9 (100.9 in control)	7.9×	
*Zea mays* L.	Cd	Cd concentration before the start of the experiment was 6.53 mg/kg	NTA (3 mmol/kg)	≈6.71 (2.55 in control)	2.6×	[250]
*Panicum virgatum* L.	Pb	Highly contaminated soil from the former Superfund site in Cedartown, GA. Pb concentrations (up to 260,000 mg/kg)	NTA (1 mM)	122 (82 in control)	≈1.5×	[263]
*Indocalamus decorus Q. H. Dai*	Pb	Soil mixed with CH_3_COOH)_2_Pb·3H_2_O to obtain contaminated soil with a Pb concentration of 1500 mg/kg	NTA (1500 mg/kg soil)	≈500 (420 in control)	1.2×	[264]
*Solanum nigrum*	Cd	Soil spiked with different Cd treatments (12 and 48 mg/kg soil)	NTA 10 mM	7.63 (4.08 in +Cd12)	1.9×	[15]
			NTA 10 mM	25.08 (16.11 in +Cd48)	1.6×	
*Zea mays* L.	Cd	Cd concentration before the start of the experiment was 6.53 mg/kg	AES (3 mmol/kg)	≈3.90 (2.55 in control)	1.5×	[250]

Footnotes: 1: Shoot heavy metal concentrations and enhancement factors were estimated from published figures using visual digitization; values represent approximate means. 2: Enhancement factor (EF) was calculated by the formula (EF = metal concentration in foliar plant part treated with chelate/metal concentration in foliar plant part without chelate). 3: EDTA = Ethylenediamine tetraacetic acid; NTA = Diethylenetriacetic acid; GLDA = Tetrasodium N, 9 N-diacetate; AES = Aspartate dibutyric acid ether, and IDSA = Iminodisuccinic acid; EDDS: Ethylenediamine disuccinate.

**Table 7:** Effects of Phosphorus Nutrition on Arbuscular Mycorrhizal Fungi (AMF) on Heavy Metal Detoxification in Host Plants.

AMF Species	Host Plant	Heavy Metal	Heavy Metal and P Fertilization Effect of Arbuscular Mycorrhizal Fungi	AMF/P Fertilization Effect on Plants	Heavy Metal Detoxification Mechanism	Citation
*Glomus intraradices*	*Zea mays* L.	Zn, Cu, Mn and Fe	-Mixture of the heavy metals at high dose reduce the hyphal length from 1.33 to 1.07 mg/g soil -Increasing P from 10 to 60 mg/kg decreased the extraradical hyphal length from 1.63 to 1.33 mg/g soil	-AMF and P fertilization increased plant biomass	-At the high level of micronutrients, there was less Mn and Fe uptake by AMF-colonized plants than by non-mycorrhizal plants -AMF plants grown with high levels of micronutrients had neither higher nor lower Cu and Zn contents in shoots	[317]
*Glomus* * mosseae*	*Trifolium pratense*	Zn	-Increasing Zn from 0 to 400 mg/kg reduced the colonization from 35% to 13.9% -Increasing P from 0 to 100 mg/kg reduced the AMF colonization 35% to 22%	-Shoot yields were highest in AM plants with added P	-AMF significantly reduced the shoot Zn concentration at 400 mg/kg applied Zn -Soil solution pH was higher in AMF treatments, and soil solution Zn was lower in the presence of mycorrhiza.	[318]
Mycorrhizal inoculum was collected from the rhizosphere of basin wildrye	*Leymus cinereus*	As	-Root AMF colonization was 29% in As 50 mg/kg+ P 15 mg/kg P treatment while minimal colonization (5%) was observed with As 3 mg/kg + P 3 mg/kg	-AMF did not influence biomass production -P application increased the shoot biomass and decreased the root biomass	-Arsenic was sequestered in the roots with 30 to 50 times more As in the roots than shoots under low P	[319]
*Glomus intraradices*	*Daucus carota* L.	^233^U	Hyphae length and number of spores developing in the root compartment were 1075 cm and 4726, respectively	-	-Hyphal tissues limit the uptake and translocation of U, -Hyphae sequestered U and reduced its translocation compared to P. -High pH in hyphal compartment	[320]
*Glomus* * mosseae*	*Cucumis sativus*	Cu, Zn, Cd and Ni	Elevated levels of metals (50 mg Cu, 100 mg Zn, 1 mg Cd, and 25 mg Ni) reduced root colonization from 70% to 67.5%. -Increasing P from 10 to 100 mg/kg soil reduced root colonization 75 to 70%	-AMF increased shoot and root biomass production -P fertilization did not have any effect on the shoot and root biomass	-AMF decreased shoot of Cd and Ni concentration while, shoot Zn and Cu concentrations were increased. -AMF improved selectivity of plant-essential elements over non-essential -Metal accumulation in the root may all contribute to the successful growth of mycorrhizal plants on metal-rich substrates.	[321]
*Glomus mosseae*	*Zea mays* L.*)*	Cd and Zn	-Cd (100 mg/kg soil) reduced root colonization from 41% to 21%, While Zn (900 mg/kg soil) reduced colonization 56% to 46%	-AMF plants had higher biomass -AMF inoculation increased plant growth with enhancement of P nutrition under P deficiency	-AMF increased plant biomass, diluting the metals in the plant tissues, -Increased soil pH in the rhizosphere and making the metals less available for plant uptake, -Reduced the concentration of soluble Zn and Cd in the soil solution Enhancing P nutrition -Decreased Cd translocation from roots to shoots,	[134]
*Glomus mosseae*	*Zea mays* L.	As	-P fertilization (100 mg/P kg soil) reduced the root infection rate (26.2%) and hyphal length density (1.8 mg/g) compared to No P added treatment with infection rate 51.6% and hyphal length density of 4.5 mg/g	-AMF increased root length and dry weight under the No-P treatments -P application decreased shoot and root biomass in AMF plants	-AMF induced plant resistance to As contamination by decreasing As translocation from roots to shoots and by the preferential uptake of P over As, and enhancing host plant growth in soils with low-P status	[322]
Glomus mosseae	*Medicago sativa Linn*	As	As reduced hyphal length density from 7.8 mg/g in 0 mg/kg As to 4.8 mg/g in 100 mg/kg As -At higher P level hyphal development was higher	-Fungal colonization increased plant dry weight by a factor of around 6, and also substantially increased both plant P and As contents	-AMF reduced As concentrations, compared to uninoculated controls. The decreased shoot As concentrations were largely due to “dilution effects” that resulted from stimulated growth of AM plants and reduced As partitioning to shoots	[323]
*Glomus* * aggregatum*	*Helianthus annuus* L.	As	-Soil containing As 620 mg/kg amended with No P, inoculated with AMF increased mycorrhizal colonization from 7.5 to 44.5% while soil with 30 mg/kg P AMF inoculation increased colonization was from 5.7 to 39.6%	-Plant shoot growth was highest in the AMF treatment -P fertilization improved root length and shoot biomass	-AM inoculation could be explained by the enhancement of P nutrition without a concomitant increase in As contents -AMF transformed inorganic As species into less toxic methylated forms, i.e., DMAA	[324]
*Glomus intraradices*	*Linum usitatissimum* L.	Cd	Cd addition did have effect on root colonization	-AMF inoculation increased the shoot growth ranging from 3.3% to 78.5% -No P-fertilization effect on plant shoot biomass was observed	-Inoculation with *G. intraradices* decreased shoot Cd at low Cd level, which was attributed to reduced root-to-shoot Cd translocation. -In contrast, *G. intraradices* inoculation increased shoot Cd at high Cd addition, which might be associated with the greater absorption of Cd by extraradical hyphae and decreased rhizosphere pH.	[325]
Indigenous AM fungal spores were isolated from the rhizosphere soil of *V. baoashanesis* collected from the Baoshan Lead/Zn Mine	*Viola baoshanensis*	Cd	Cd applications of 50, 100 and 200 mg/kg soil decreased the ratio of fragments with arbuscules or hyphae to the total fragments around 17, 14 and 45%, respectively	-AMF colonization increased shoot biomass at low P levels, and this beneficial effect was diminished by high P availability -Increasing P resulted in higher shoot biomass.	AM colonization decreased shoot Cd concentrations. At low Cd bioavailability, reduced Cd uptake was due to decreased Cd translocation from the roots to the shoots, whereas at high Cd reduced root Cd uptake attributed to decreased plant Cd contents	[326]
		Pb	Pb applications of 500, 1000 and 1500 mg/kg soil decreased the ratio of fragments with arbuscules or hyphae to the total fragments around 15, 33 and 41% with, respectively		-Root phosphorus concentrations could play a role in Pb translocation from the roots to the shoots, due to intracellular precipitation of metallic cations with PO_4_, as seen in the negative correlation between shoot Pb and root P concentrations.	
*Glomus mosseae*	*Medicago sativa* L.	Cd, Co, Pb	Inoculation increased the Mycorrhizal colonization to 48% compared to non-inoculated control with only 21%	-AMF increased the shoot dry weight and shoot P concentration *-G. mosseae* decreased the shoot Cd, Co, and Pb concentrations	-AMF might have selectively contributed to the exclusion of toxic and nontoxic elements (i.e., myco-rhizo-remediation). Metals may be sequestered in the hyphae and not translocated to the plants	[327]
*Rhizophagus irregularis*	*Trifolium ripense* L.	Cd	-1 mM Cd decreased root colonization by from 75% to 56% -Increasing P from 0.1 mM to 1 mM decreased root colonization from 68 to 56% under 1 mM Cd stress -Under 0 mM Cd P has no effect on AMF colonization	-No P-fertilization effect on plant shoot and root growth -P increased polyP accumulation in hyphare. PolyP accumulation enhanced AM resistance to Cd, and also reduced Cd uptake by plants.	-P application can enhance the resistance of AMF to Cd *via* polyP chelation	[328]
*Rhizophagus irregularis* (formerly Glomus intraradices) and *Funneliformis mosseae* (formerly Glomus mosseae)	*Cucurbita pepo* L.	Al and low pH	-Low pH significantly reduced root colonization. -At pH 6 root colonization was 48% that reduced to 23% at pH 3.5 and it further reduced with pH 3.5 +Al to 17%	-Both inoculants increased yield and no. of marketable fruits	-At low pH AMF increased the P concentration while reduced the Al in leaves and fruits -Higher SPAD value, and cell membrane stability alleviated the impacts of acidity and Al toxicity	[329]
*Gigaspora margarita (GM)* *Acaulospora longula (AM) and* * Rhizophagus irregularis (RI)*	*Lotus japonicus* L.	Cd	-Cd at 50 mg/kg reduced root colonization around 9% *Gigaspora margarita*, *44% in Acaulospora longula and 21% in Rhizophagus irregularis*	-Generally, both shoot and root dry weights were increased by mycorrhizal colonization -AMF inoculation showed a different response to Cd in the root length-specific P uptake efficiency.	-P uptake efficiency generally determined the host growth and the Cd uptake of the symbiotic associations under Cd contamination. *-R. irregularis* was more resistant to Cd contamination compared with the other two AM fungal species in terms of higher colonization rate, P uptake efficiency and better plant growth.	[330]
*Gigaspora* * margarita*, * Rhizophagus irregularis or Glomus claroideum*	*Glycine max*	Al	-At low P (LP), mycorrhizal growth response (MGR) was about 45% for *Gigaspora margarita*, 22% for *Rhizophagus irregularis*, and 30% for *Glomus claroideum* without Al, which changed to approximately 65%, 12%, and 30%, respectively, under 600 µM Al. -At high P (HP), MGR under –Al was very low (~6–12% for all species), but increased markedly under 600 µM Al to about 50% in *Gigaspora margarita*, 58–60% in *Rhizophagus irregularis*, and 60–62% in *Glomus claroideum*	-All the inoculates increased the shoot biomass, while the root biomass was remained un-changed	-AMF improved P nutrition -High P availability reduces Al toxicity -AMF decreased the root Al concentrations -AMF increase in the expression of the P-transporter gene *GmPT9* in soybean	[331]
*Funneliformis caledonium* (Fc) or *Glomus versiforme* (Gv).	*Cucumis sativus* L.	Cd contaminated soil with total Cd 1.6 mg/kg	-AMF inoculation increased mycorrhizal colonization compared to non-inoculated control	-AMF increased shoot biomass production	-AMF association with Fc and Gv decreased root and shoot Cd concentrations, with increased P/Cd ratios, -Only Gv significantly decreased the root Cd acquisition efficiency and increased the root-to-shoot Cd translocation factor. -AMF also decrease soil total and phytoavailable Cd concentrations by increasing plant total Cd acquisition and soil pH -AMF increased acid phosphatase activity	[332]
*Glomus intraradices*	*Zea mays* L.	Soil irrigated with sewage for 40 years contaminated with Fe, Mn, Zn, Cu, Cd and Pb	Phosphorus decreased; Spore density: 85 to 48/g Hyphal colonization:73 to 42%/ Vesicular colonization:24 to 12% Arbuscular colonization: 48 to 25%	-P fertilization at 30 mg/kg, with *G. intraradices* increased shoot biomass -P-fertilization negatively affected *G. intraradices colonization*	*-G. aggregatum* inoculation decreased shoot concentrations of Cd, Pb and Zn chelation/immobilization of metals by glomalin, or exudates -Diluting the concentration of heavy metals in the shoot with the increase in growth	[135]
*Glomus aggregatum*			Phosphorus decreased; Spore density: 74 to 38/g Hyphal colonization: 62–35% Vesicular colonization: 20 to 10% Arbuscular colonization: 42 to 20%	-No positive effects P fertilization at 30 mg/kg, with *G. intraradices* on shoot biomass -P-fertilization negatively affected *G. aggregatum* colonization	*-G. intraradices* inoculation increased Cd and Pb concentration in maize shoot	
AMF inoculum include the consortia of *Glomus aggregatum*, *G. intraradices*, *G. elunicatum* and *G. versiforme*	*Lolium multiflorum*	Cd	Root colonization was not determined	-P fertilizer increased the biomass and N uptake -Mycorrhizal inoculation had no positive influence on the plant shoot biomass and N, P, K, Ca and Mg in plants	-AMF reduced the Cd translocation from roots to shoots	[333]
*Funneliformis mosseae*	*Glycine max*	Pb	Pb decreased root length colonization from 65% to 44% at 300 mg Pb/kg soil	-AMF inoculation promoted plant growth, P uptake -Decrease in P uptake was observed with the Pb addition in the soil	*-R. intraradices* was more tolerant to Pb stress than other *F. mosseae* and *C. etunicatum* in terms of higher root colonization, -P uptake strongly determines the soybean growth and Pb uptake in Pb-contaminated soils. -Increased biomass, P uptake and retention of Pb in the roots with AMF inoculation promoted soybean growth under Pb contamination	[334]
*Claroideoglomus etunicatum*			Pb decreased root length colonization from 55% to 42% at 300 mg Pb/kg soil			
*R. intraradices*			Pb decreased root length colonization from 72% to 42% at 300 mg Pb/kg soil			
*Rhizophagus* * intraradices*	*Zea mays var. Weike6*	Ag and Ag-nanoparticles	Ag decreased ≈10% Root mycorrhizal colonization while AgNPs decreased it ≈ 35%	-P increased both shoot and root biomass of non-inoculated plants, -AMF inoculation produced positive impacts on plant biomass, nutritional and physiological responses	-P application retained Ag was detected in the roots of non-mycorrhizal plant. -Plants inoculated with AMF or receiving P generally had much higher photosynthesis performance, and thereby improve the efficiency of PSII photochemical activity.	[335]
Native mycorrhizal fungi	*Triticum aestivum* L. *cv.* * Tahirova*	Cd	-P fertilization reduced AMF colonization	-P increased shoot growth -Root mycorrhizal colonization showed significant negative effect on shoot Cd content	-Root mycorrhizal colonization showed a statistically highly significant negative relationship with the shoot Cd content Mycorrhizae role to reduce root Cd uptake is related to their role in increased root Zn uptake.	[336]
*Funneliformis mosseae*	*Phragmites australis*	Cd	-Cd decreased the Colonization rate Increasing P decreased the colonization rate	-AMF increased the shoot and root biomass and Cd tolerance index -P increased the shoot and root biomass	-AMF promoted plant Cd tolerance and detoxification by enhancing P uptake, Cd passivation, Cd retention in the cell wall, and functional group modulation. -Cd was mainly present in the cell wall fraction (41.75– 74.06%) -AMF up-regulated several *HMA2* genes in the high P treatment, which reduced cellular Cd toxicity -At limited P, higher Cd was retained in stem, protecting the leaves	[337]
*Rhizophagus intraradices*	*Eucalyptus tereticornis*	Mn	-Mycorrhizal frequency was highest in control treatment under low P availability (80%), decreased to approximately 40% after Mn treatment and P supply	-AMF improved the growth -P-fertilization improved the biomass	-Mycorrhizal symbiosis did not influence Mn accumulation, but improved growth and Mn tolerance at low P, partly by improving P nutrition -AMF downregulated most of the Mn- influx transporters, i.e., *EtVIT1* and *EtMTP8* and the Mn-nicotianamine influx transporter *EtYSL6*.	[338]
*Rhizophagus irregularis*	*Broussonetia papyrifera*	Cd (0, 30, 90 and 270 mg/kg)	Cadmium significantly affected AMF colonization in *B. papyrifera*,—total colonization slightly increased from 0.73 (Cd0) to 0.788 (Cd30) but declined to 0.655 (Cd90) and 0.393 (Cd270). -Hyphae decreased from 0.438 (Cd30) to 0.262 (Cd270), arbuscules from 0.195 (Cd0) to 0.057 (Cd270), and vesicles from 0.098 (Cd30) to 0.013 (Cd270), indicating progressive inhibition of AMF structures with increasing Cd stress.	-AM symbiosis improved the growth and photosynthesis,	-AMF enhanced ROS levels as stress signaling and maintained ROS balance under low and medium Cd stress. -AMF symbiosis regulated AsA-GSH cycle to mitigate ROS overproduction under high Cd stress. -AMF chelate more Cd under high Cd stress, increasing soil pH and glomalin soil protein content. -AMF plants can fix or chelate more Cd by P in leaves and reserve more P in stems under high Cd stress. AMF symbioses increased root net Cd^2+^ influx and uptake under medium Cd stress but inhibited under high Cd stress, with upregulation of genes related heavy metals (HMs) transport under medium Cd stress and inhibited the transcription of genes related HMs transport under high Cd stress.	[339]
*Funneliformis mosseae*	*Trifolium repens* L.	Al	-Al stress decreased the AMF colonization from 61.9 to 49.8%, -Al stress decreased Hyphal length from 1822 to 1085 mm/g soil and Arbuscules from 4.51 to 3.60 (number/cm root)	-AMF increased the plant biomass accumulation -AMF increased root length	-AMF reduced Al accumulation and increased P content in the roots -AMF decreased ROS (H_2_O_2_, and O_2_^−^) by SOD, POD, and CAT and AsA-GSH cycle in the leaves and roots -AMF upregulated of genes involved in P transport (*PHO1-2* and *PHT1-7*), the AsA-GSH pathway (*GST-2* and *APX-2*), and Al stress (*ALMT1*)	[340]
*Funneliformis* * mosseae*	*Phragmites australis*	Cd	-Higher colonization rate was observed at high Cd and medium P application	-AMF increased the shoot biomass -Increasing P application reduced shoot biomass	-Under Cd stress, P addition alleviated the harmful effects of Cd by increasing proline accumulation -AMF and P alleviated Cd-induced lipid peroxidation and H_2_O_2_, O_2_^.−^ accumulation through anti-oxidative enzymes and secondary metabolites -AMF-induced auxin, cytokinine, jasmonate and brassinosteroid signaling pathways	[341]
*R. irregularis* BGC JX04B	*Zea mays* (M191 and M265) with P–Zn antagonistic interaction and two genotypes (M259 andM340) without P–Zn antagonistic interaction	Zn	-Antagonistic genotypes displayed a notably greater decrease in AM colonization (93%) and hyphae length density (83%) under high P treatment compared with non-antagonistic genotypes, which showed a reduction of 60% in AM colonization and 53% in hyphae length density	-Increased soil P availability inhibited the colonization rate of AM fungi in the root and the hyphae length density in the soil.	-Maize genotypes without P–Zn antagonistic interaction exhibited higher levels of AM colonization and hyphae length density compared with genotypes with P–Zn antagonism under high P treatment. -High P treatment reduces Zn uptake by extraradical hyphae, potentially by suppressing the expression of *RiZRT1* and *RiZnT1*.	[342]
*Funneliformis* * mosseae*	*Phragmites* * australis*	Cd	-Colonization was not determined	-AMF increased shoot biomass at no and low Cd levels -AMF increased the P concentration in plants	-AMF increased Cd resistance through mechanisms, including improved rhizosphere nutrient availability and increased proline accumulation	[343]

**Table 8:** Effect of plant-growth-promoting rhizobacteria and organic fertilizer on heavy metal-remediation.

Plant Species	Heavy Metal	Plant Growing Conditions and Amendment	Microbe Involved	Mechanism of Remediation	Citation
*Trifolium repens*	Zn	Soil/sand mixture was supplemented by Zn, in order to have final concentration of 600 mg/kg. It was amended with *Aspergillus niger*-treated sugar beet waste (SBW, 50 g/kg soil),	*Glomus mosseae*	-Increased shoot biomass, N, P and K nutrition and decreased Zn acquisition -Improved nodule formation -Enhanced activities of phosphatase, dehydrogenase and β-glucosidase activities	[353]
*Trifolium repens*	Fe, Mn, Al, Zn, Pb, Cu, Cd, Ni, As and Cr	The multi-contaminated test soil (Gorguel) selected from Murcia province (Spain) sugar beet waste, containing Fe (139,045 mg/kg), Mn (8300 mg/kg), Al (19,385 mg/kg), Zn (47,695 mg/kg), Pb (8555 mg/kg), Cu (168 mg/kg), Cd (52 mg/kg), Ni (34 mg/kg), As (475 mg/kg) and Cr (31 mg/kg), was amended with fermented sugarbeet waste	Arbuscular mycorrhizal fungi and *Bacillus cereus*	-Organic amendment with AM inoculation increased the microbial diversity by 233% and changed bacterial community structure (by 215%) -The microbial inoculants and amendment favored plant growth and the phytoextraction process	[360]
*Brassica juncea*	Cd	Soil spiked with Cd (100 mg/kg) was amended with 2% (*w*/*w*) FYM vermicompost	*Azotobacter* sp. and *Pseudomonas* sp.	-Amendments increased the shoot and root Cd accumulations without any significant impact on plant growth	[361]
*Tetraclinis articulata and Crithmum maritimum*	Pb, Zn and Al	Mine tailing called “El Gorguel”, containing Pb (6.9 g/kg), Zn (12 g/kg), andAl (14.5 g/kg), was amended with fermented sugar beet residues (1.2% *w*/*w*).	*Glomus mosseae*,	-Amendments increased biomass in *T. articulata* while there was no effect on *C. maritimum* -P contents in plants were increased, root colonization was increased in *C. maritimum* -In the rhizosphere, phosphatase, β-glucosidase and dehydrogenase activity was increased	[354]
*Trifolium repens* L.	Zn, Cd and Pb	Soil was collected from the mining area located in the Cartagena–La Union Mining District (southeastern Spain), containing Zn (27.5 g/kg), Cd (34 mg/kg), Pb (6.43 g/kg), Mn (5.4 g/kg), and Ni (26 mg/kg), was mixed with sand (1:1) and the vermicomposts	*Rhizobium leguminosarum bv. trifolii*	-Plant biomass was increased. Likewise, the P, K, Fe, Mn, Cu and Zn uptake by the plant increased -AB-DTPA-extractable Pb, Cd and Zn in the postharvest soil were decreased -Dehydrogenase, β-glucosidase, and urease activities in the postharvest soils were increased with the amendments, while the phosphatase activity was decreased	[355]
*Tetraclinis articulata*	Pb, Zn, Al	Soil sampling was conducted at one mine tailing called “El Gorguel, containing Pb (6.9 g/kg), Zn (12 g/kg), and Al (14.5 g/kg), was amended composted olive waste @ 1% (*w*/*w*) and 3% *w*/*w*)	*Glomus mosseae*	-AMF and composted olive waste increased shoot and root biomass production of *T. articulata* by 96 and 60%, respectively -Decreased the Cr, Ni, and Pb contents in shoot, as well as Cr and As in root -Improved nutrient uptake, mainly P and soil microbial function -Enhanced activities of rhizosphere enzymes dehydrogenase, urease, protease, phosphatase, and β-glucosidase	[362]
*Oenothera picensis*	Cu	Soil and sand mixture (2:1 soil:sand, *v*/*v*), 300 mL pots and supplemented with 0, 100, or 500 mg Cu/kg. Later it was amended with biotreated sugar beet agro waste (5%, *w*/*w*)	*Claroideoglomus claroideum*	-Amendments (AMF and sugar beet agro-waste) produced a higher plant growth -Improved plant survival at highest copper concentrations -At higher Cu concentration, AMF + sugar beet agro waste reduced root and shoot concentration and decreased translocation	[363]
*Raphanus sativus*	Pb	Soil spiked with 500 mg/kg soil amended with amended compost was used for the experiment	*Bacillus* sp. *CIK-512*	-Amendments (compost + *Bacillus* sp.) improved root and shoot biomass -Improved antioxidant capacity and membrane stability -Decreased translocation of Pb from root to shot	[364]
*Oryza sativa*	Cu	Field trial with compost application on rice (0, 1, 2, 4 kg/m^2^) Mine-affected fields have high levels of soil Cu (281 mg/kg)	*Trichoderma microbial inoculum*	-Amendments increased productive tillers in plants and grain weight -Increase in soil pH was observed	[365]
*Brassica juncea*	Zn, Cd and Pb	Greenhouse experiment was conducted using a metal-polluted soil (total metal concentrations Zn = 285, Cd = 2.95, and Pb = 95 mg/kg) amended with *Sesbania* green manure	*Pseudomonas* spp. *PS2-79*	-Increased the root, shoot and grain biomass -Amendments decreased the soluble metal in the soil and increased the organic bound soil fraction -Increased the metal concentrations in shoot	[358]
*Helianthus annuus*	Pb	Soil was contaminated (600 mg/kg) by Pb (NO_3_)_2_ and composted with pressmud 2% (*w*/*w*) as an organic amendment	*Pseudomonas gessardii BLP141*	-Improved the plant growth (65%), total chlorophyll contents (63%), yield (47%) -Pb immobilization (78%) over control in Pb polluted soil	[366]
Wild olive (*Olea europaea* var. sylvestris) and stone pine (*Pinus pinea*).	As, Cu, Pb, Zn	Field experiment was carried out at the Vicario experimental site in the Guadiamar Green Corridor, contaminated by trace elements (As 171 mg/kg, Cu 78.8 mg/kg, Pb 320 mg/kg and Zn 159 mg/kg) after a mine spill in 1998. Soil was amended with 30 and 60 T/ha of equal mix of biosolid compost (BC) and sugar lime (SL)	*Rhizophagus irregularis (*formerly *Glomus* * intraradices)*	-No positive effect of the mycorrhizal inoculation on plant growth, survival, and trace element accumulation. -Soil amendments with compost were effective in reducing trace elements availability and their accumulation in both plant species, especially in roots -Both plants species, wild olive and stone pine, have shown to be adequate for phytostabilization of soils contaminated by trace elements	[367]
*Solanum* * lycopersicum* L.	Cd, Ni, Cr, Pb, and As	Pots experiment with soil containing Cd (0.76 mg/kg), Ni, 8.72 mg/kg) Cr (4.18 mg/kg), Pb (6.22 mg/kg), and As (3.18 mg/kg) amended with farm yard manure	*Serratia marcescens—SF3*	-Improve plant growth and yield characteristics of tomato were observed -Amendment and PGPR reduced heavy metal concentration in root and shoot by immobilizing the metal in soil	[368]
*Solanum* * lycopersicum* L.	Cd	Growth medium containing Cd @ 2 mg/kg was amended with 0.5% of organic amendments (compost and biochar) were mixed with sand before transplanting tomato seedlings	*Serratia plymuthica J-62* *Pseudomonas aeruginosa J-156*, *J-159* *Serratia marcescens J-165*	-Increased plant biomass by IAA production -Amendments decreased plants Cd concentrations	[369]
*Medicago sativa* L. and *Solanum nigrum* L.	Cd, Zn and Pb	Soils were collected from a steel industrial area located in the city of Jinan, China (36°43′ N; 117°10′ E). The mill was closed for relocation in 2017 after producing steel for nearly 60 years.	Microbial inoculant	-*S. nigrum* L. removed higher amounts of Cd than Zn and Pb, while *M. sativa* L. uptake more Zn. -Enhanced antioxidant defense systems (SOD, POD, CAT) reduced membrane damages Soil enzymes dehydrogenase, urease, and catalase increased up to 5-fold. -Soil bacterial diversity and structure was changed, being largely composed of Proteobacteria, Actinobacteria, Patescibacteria, Bacteroidetes, and Firmicutes.	[359]
*Solanum lycopersicum*	Cd	Soil was contaminated with Cd (2 mg/kg) and amended with 0.5% *w*/*w* of compost and biochar along with rhizobacteria n pot culture	*Pseudomonas gessardii strain BLP141)*, *(Pseudomonas fluorescens A506)* and *(Pseudomonas fluorescens strain LMG 2189*	-Improved root and shoot lengths (112 and 72%), fresh (130 and 146%) and dry weights (119 and 162%), respectively -Improved antioxidant capacity -Combined application of ‘J-62’ strain and organic amendments decreased Cd translocation	[370]
*Spinacia oleracea*	Cr	Soil spiked with Cr (16 mg/kg) was amended with compost (1% *w*/*w*) prepared from rice straw	Cr-resistant rhizobacterial	-Amendment and PGPR improved plant biomass -Improved antioxidative enzyme activities -Decreased Cr translocation and phytostabilized Cr in spinach rhizosphere	[371]
*Vigna radiata* L.	Cr	Soil was contaminated with K_2_Cr_2_O_7_ @ 5, 10, and 15 mg/kg amended with Biogas slurry at the rate of 800 kg/ha	*Rhizobia strains*, i.e., HQ5	-Combinedly PGPR and Biogas slurry at 15 mg Cr/kg increased plant height (42%), shoot fresh (88%), and dry weights (1.42-fold), root length (77%), number of pods plant 1 (2.54-fold), grain pod 1 (1.36-fold), pods fresh (1.58-fold) and dry weights (1.26-fold) -Improved antioxidant capacity and maintained membrane integrity -Decreased Cr in plant root and shoot	[357]
*Lygeum spartum.*	Zn, Pb, Cu, Cd, Ni and Mn	Mine tailing consisting of total metal concentrations in mg/kg: Zn= 6738, Pb = 4658, Cu = 234, Cd =37, Ni = 31 and Mn =4421 was amended with fermented sugar beet residues (5% weight/weight)	*Funneliformis mosseae and Bacillus cereus*	Shoot and root biomass was increased of about 410% and 370%, respectively Increased root heavy metal content Decreased root acid phospho-mono-esterase activity. -Increased soil dehydrogenase, protease and β-glucosidase activities, total N content and aggregate stability	[372]
*Populus nigra* L. planted alone (P) or in co-cropping with *Trifolium repens* L.	Al, Mn, Cu, Cr, Ni, Cd and Co	Soil from the mine tailing deposit of a former copper mine with the pseudo-total metal concentrations Al (42.5 g/kg), Mn (823 mg/kg), Cu (596 mg/kg)), Cr (96 mg/kg), Ni (43 mg/kg) Cd (1.6 mg/kg), and Co (12.2 mg/kg) was used in this study. To prepare the substrate for the pot experiment, over 100 kg of mine soil material was mixed with compost at a ratio of 1:10 (*w*/*w*)	*Bacillus* sp. *SK2.3* (IAA producer), *Rhodococcus* sp. *SK12.6* (IAA and biosurfactant producer, and inorganic phosphate solubilizer), *Streptomyces* sp. *SK20.12* (IAA and siderophore producer), and *Massilia niastensis P87* (IAA producer) *Mycorrhizal* inoculum consisted of a commercial mixture by the company INOQ GmbH, Germany	-Bio-inoculum increase in plant survival and development due to IAA production by inoculant, biosurfactants and siderophores, and to solubilize phosphate -Amendments increase the soil pH and decrease Al and Cu availability -Soil bacterial functional diversity was markedly influenced by the presence of plants and the inoculation with bacteria	[356]
*Helianthus annuus* L.	Pb	Pots experiment with soil spiked with 500 mg/kg soil was used to grow sunflower. The organic amendment used for the experiment was compost (1% in soil)	*Pseudomonas fluorescens and Pseudomonas gessardi’*	-Pb plant uptake and its phytostabilization in roots reduced metal mobility to shoot and contributed in better plant performance -The efficient strain *Pseudomonas fluorescens* along with compost increased the plant N, P, and K -Flavonoid and phenolic contents were increased with improved antioxidant capacity	[373]
*Aloe barbadensis*	Pb, Ni and Cd	Soil with a history of industrial wastewater application containing available Cd 0.96 mg/kg, Ni 1.95 mg/kg and Pb 11.62 mg/kg was amended with pressmud (2% *w*/*w*)	*Pseudomonas fluorescens*	-Combined amendment application increased the plant biomass -Improved antioxidant system -Improved plant nutrition (N, P, K Ca concentrations) -Decreased Cd, Ni and Pb concentrations in plant	[374]
*Lolium perenne* L.	Cd	Soil contaminated with Cd (2.2 mg/kg) was amended with rice straw, wheat straw, and cow dung (1% *w*/*w*)	*Pseudomonas fluorescens*	-Combined amendments application increased the plant biomass -Higher Cd retention in roots	[375]
*Zea mays*	Ni and Zn	HM polluted soils, Ni (150 mg/kg) and Zn (300 mg kg*^−^*^1^), were artificially spiked in the sterilized garden; soil was mixed with groundnut shell biochar (5% *w*/*w*)	*Bacillus pseudomycoides* strain ARN7	*-Bacillus pseudomycoides* strain ARN7 and groundnut shell biochar improved plant growth -Increased chlorophyll, proteins, phenolics contents and SOD, APX, and CAT, -Reducing electrolyte leakage and MDA, proline, and metal accumulation and bioaccumulation factor -Soil dehydrogenase, urease, alkaline phosphatase, and β-glucosidase activities were improved	[376]
*Sorghum bicolor* (L.)	Cd and Zn	Soil contaminated with Cd and Zn was amended with banana pith-derived biochar (0, 2.5 and 5%, *w*/*w*)	*Bacillus thuringiensis* SE1C2	-Combined treatment of BC 5% and SE1C2 increased plant shoot length, root length, fresh weight and dry weight but decreased Cd and Zn accumulation -Chlorophyll, carotenoid and antioxidant enzymes were improved with lower leaf malondialdehyde and proline contents -Improved enzymatic activities (acid phosphatases, alkaline phosphatases, β-glucosidase and urease)	[377]
*Triticum aestivum*	Cd	Corn stalk and farmyard manure biochar	*Bacillus cereus* and *Pseudomonas frederiksbergensis*	-Plant growth was improved -Increase in osmo-protectants and antioxidants activity was observed -Cd uptake in roots and shoots decreased up to 39.37 and 55.32%	[378]
*Zea mays*	Cr	Greenhouse pot experiment was conducted to investigate the effect of Cr along with biochar of different feedstocks	*Bacillus subtilis* and *Pseudomonas aeruginosa strains*	-Improved root-shoot length (36 and 10% respectively), total chlorophyll (11.29%), and stomatal conductance (11.95%). -Carotenoid, flavonoid, and phenolic contents with enhanced antioxidants superoxidase, peroxidase, and catalase activities were observed	[379]
*Oryza sativa*	Pb and Al	Soil with total Pb and Al after soil aging contains 380.42 mg/kg and 365.12 mg/kg, was amended with corn straw biochar (1.5 g/400 g soil)	*Bacillus amyloliquefaciens* SQ-2	-Activities of CAT and SOD increased by 162.5% and 162.9%, respectively. -Phenolic and chlorophyll contents of rice increased by 17.6% and 83.7%, respectively -Soil nutrient contents were improved, i.e., nitrate N, available K, and sucrase were enhanced by 9.4%, 45.9%, and 466.8%, respectively -Biosorption of Pb^2+^ and Al^3+^	[20]
*Cicer arietinum*	Pb, Cd	Contaminated soil receiving sewage and factory wastewater over the last 60 years amended with press-mud	Consortia of metal-tolerant (Pb and Cd) rhizobacterial and rhizobial strains	-Decreased Pb and Cd concentrations -Increased shoot fresh weight, root fresh weight, root length, no. of nodules and photosynthetic rate	[380]
*P. vittata*	As	Arsenic-contaminated soil (250 mg/kg As) was amended with corn stalk biochar (BC). Through pot experiments, effects of various BC addition rates (0%, 1%, 5%) on plants were determined	*Burkholderia contaminans ZCC*	-Increased plant biomass (2.56 times that of the control) and total chlorophyll content (43.32% higher) -Increased SOD, POD, and CAT activities -Reduced MDA content, improved osmotic regulation through higher proline content	[381]

## Data Availability

The original contributions presented in this study are included in the article/Supplementary Material. Further inquiries can be directed to the corresponding author.

## Supplementary Materials

The following supporting information can be downloaded at https://www.mdpi.com/article/10.3390/plants15101517/s1; Table S1: Effects of heavy metal stress on arbuscular mycorrhizal fungi (AMF) colonization and hyphal development across different metals and experimental conditions. The table summarizes reported reductions in AMF structural parameters including hyphal length, root colonization, arbuscules, vesicles, and mycorrhizal frequency under Cd, Zn, Pb, Cu, As, Mn, Al, and Ag/Ag-NP stress; Table S2: Decision-support framework linking heavy metals, remediation strategies, chelator-assisted phytoextraction, and nutrient management.
