# Supplementary material for: Nutrient-Driven Modulation of Microbial, Plant, and Rhizosphere Processes for Heavy Metal Remediation
Source: Plants (Basel). 2026 May 15;15(10):1517. doi: 10.3390/plants15101517 (PMC13210518; doi:10.3390/plants15101517)
Supplement: Supplementary file 1 [file plants-15-01517-s001.zip › plants-4297408-supplementary.pdf]

Table S1: Effects of heavy metal stress on arbuscular mycorrhizal fungi (AMF) colonization and hyphal development across different metals and experimental conditions. The table summarizes reported reductions in AMF structural parameters including hyphal length, root colonization, arbuscules, vesicles, and mycorrhizal frequency under Cd, Zn, Pb, Cu, As, Mn, Al, and Ag/Ag-NP stress.

Parameter	Heavy metal stress	Control value	Treatment value	Percent reduction (%)	Citation
Hyphal length reduction under mixed heavy metals	Zn, Cu, Mn, Fe	1.33	1.07	19.55	[317]
Extraradical hyphal length reduction with P increase	Zn, Cu, Mn, Fe	1.63	1.33	18.40	[317]
AMF colonization reduction with Zn (0→400 mg/kg)	Zn	35	13.9	60.29	[318]
AMF colonization reduction with P (0→100 mg/kg)	Zn	35	22	37.14	[318]
Root colonization reduction under Cu/Zn/Cd/Ni stress	Cu, Zn, Cd, Ni	70	67.5	3.57	[321]
Root colonization reduction with Cd (100 mg/kg)	Cd	41	21	48.78	[134]
Root colonization reduction with Zn (900 mg/kg)	Zn	56	46	17.86	[134]
Arbuscules or hyphae	Cd (50)	100	83	17.00	[326]
Arbuscules or hyphae	Cd (100)	100	86	14.00	[326]
Arbuscules or hyphae	Cd (200)	100	55	45.00	[326]
Arbuscules or hyphae	Pb(500)	100	85	15.00	[326]
Arbuscules or hyphae	Pb(1000)	100	67	33.00	[326]
Arbuscules or hyphae	Pb(1500)	100	59	41.00	[326]
Root Colonization	Cd	75	56	25.33	[328]
Root colonization (Gigaspora margarita)	Cd	100	91	9.00	[330]
Root colonization (Acaulospora longula)		100	66	34.00	[330]
Root colonization (Rhizophagus irregularis)		100	79	21.00	[330]
Hyphal length density reduction with As (0→100 mg/kg)	As	7.8	4.8	38.46	[323]
Root colonization reduction with Pb (65→44%)	Pb	65	44	32.31	[334]
Root colonization reduction with Pb (55→42%)	Pb	55	42	23.64	[334]
Root colonization reduction with Pb (72→42%)	Pb	72	42	41.67	[334]
Root mycorrhizal colonization	Ag	100	90	10.00	[335]
Root mycorrhizal colonization	Ag-NP	100	65	35.00	[335]
Mycorrhizal frequency	Mn	100	40	60.00	[338]
Total colonization (Cd0 → Cd270)	Cd	0.73	0.393	46.16	[339]
Hyphae (Cd30 → Cd270)	Cd	0.438	0.262	40.18	[339]
Arbuscules (Cd0 → Cd270)	Cd	0.195	0.057	70.77	[339]
Vesicles (Cd30 → Cd270)	Cd	0.098	0.013	86.73	[339]
AMF colonization reduction under Al stress	Al	61.9	49.8	19.55	[340]
Hyphal length reduction under Al stress	Al	1822	1085	40.45	[340]
**Mean**					**33.49**

Percentage reduction (%) = {(Control Value−Heavy metal treatment value)/ Control Value} ×100

Table S2: Decision-support framework linking heavy metals, remediation strategies, chelator-assisted phytoextraction, and nutrient management

**Heavy metal**	**Dominant nutrient interaction**	**Recommended remediation strategy**	**Chelator-assisted phytoextraction options**	**Nutrient regime / management**	**Microbial interventions**	**Mechanistic basis**
Cd (Cadmium)	Competes with Zn^{2+} and Fe^{2+} transport; detoxified via S-containing thiols	Phytoextraction or assisted phytoextraction	EDTA, EDDS, citric acid	Balanced Zn and Fe supply; adequate S fertilization to stimulate glutathione and phytochelatin synthesis	AMF (*Rhizophagus irregularis*), PGPR (*Bacillus*, *Pseudomonas*)	Chelators mobilize Cd; sulfur promotes thiol-mediated sequestration and Cd–S complex formation
Zn (Zinc)	Interacts with Cd and Fe uptake pathways	Phytoextraction	EDDS, citric acid	Balanced P fertilization; adequate S to maintain antioxidant and thiol metabolism	AMF and siderophore-producing PGPR	Chelators mobilize Zn while microbial siderophores enhance metal availability
Pb (Lead)	Strong affinity for phosphate and sulfide	Phytostabilization or controlled phytoextraction	EDTA, EDDS	Higher P fertilization for immobilization; S amendments promote Pb–S precipitation	Sulfate-reducing bacteria, phosphate-solubilizing bacteria	Formation of insoluble Pb-phosphate and Pb-sulfide minerals reduces metal mobility
As (Arsenic)	Arsenate competes with phosphate transporters	Phytostabilization or controlled phytoextraction	Organic acids (citric, oxalic acids)	Elevated P fertilization to suppress arsenate uptake; balanced N and S for detoxification metabolism	AMF and arsenate-reducing rhizobacteria	Competitive inhibition between phosphate and arsenate transporters; thiol compounds assist As sequestration
Ni (Nickel)	Competes with Fe and Zn transporters	Phytoextraction using hyperaccumulators	EDTA, citric acid	adequate S to support thiol-based detoxification	metal-tolerant PGPR	Chelators mobilize Ni while sulfur metabolism contributes to intracellular metal complexation
Cu (Copper)	Strong complexation with organic ligands and thiols	Phytoextraction or stabilization depending on contamination level	EDDS, citric acid	Organic amendments with balanced N–P–S supply	Metal-resistant PGPR and AMF	Cu detoxification involves binding to thiol-rich peptides and sulfide complexes
Cr (Chromium)	Reduction influenced by microbial metabolism and organic C availability	Bioreduction and phytostabilization	Organic acids and biodegradable chelators	Addition of organic C and balanced S supply to stimulate microbial reduction processes	Cr-reducing PGPR (*Pseudomonas*, *Bacillus*), sulfate-reducing bacteria	Microbial reduction converts toxic Cr(VI) to Cr(III); sulfide generation can immobilize metals
